# *Snord15b* Maintains Stemness of Intestinal Stem Cells via Enhancement of Alternative Splicing of *Btrc* Short Isoform for Suppression of β‐Catenin Ubiquitination

**DOI:** 10.1002/advs.202504485

**Published:** 2025-08-30

**Authors:** Yuwei Xu, Peikang Zhang, Zhen Xiong, Yufei Lan, Hui Guo, Runyuan Wu, Cunzhen Li, Hongzhe Fan, Ying Du, Xiaoxiao Zhu, Dongdong Fan, Zhonglong Wang, Yong Tian, Zusen Fan

**Affiliations:** ^1^ Key Laboratory of Epigenetic Regulation and Intervention Institute of Biophysics Chinese Academy of Sciences Beijing 100101 China; ^2^ Faculty of Pharmaceutical Sciences Shenzhen University of Advanced Technology Shenzhen 518107 China; ^3^ University of Chinese Academy of Sciences Beijing 100049 China

**Keywords:** alternative splicing, Btrc, Ilf2, intestinal stem cells, Snord15b

## Abstract

Intestinal epithelium is derived from Lgr5^+^ intestinal stem cells (ISCs) located at the crypt base. However, how small nucleolar RNAs (snoRNAs) regulate ISC stemness remains elusive. Here, a conserved snoRNA, *Snord15b*, that is highly expressed in ISCs is identified. *Snord15b* knockout abolishes the self‐renewal capacity of ISCs and impairs epithelial regeneration. Mechanistically, *Snord15b* interacts with interleukin enhancer‐binding factor 2 (Ilf2) to recruit splicing factors, which mediates alternative splicing of Btrc to form a short isoform of Btrc, resulting in abrogation of E3 ligase complex formation for ubiquitination of β‐catenin. Subsequently, stable β‐catenin translocates into the nucleus of ISCs for activation of the Wnt/β‐catenin signaling pathway, leading to ISC stemness maintenance and intestinal regeneration. Of note, knockout of *Snord15b* or *Ilf2* increases β‐catenin ubiquitination to suppress activation of Wnt signaling. Furthermore, *Btrc* knockout blocks β‐catenin ubiquitination to enhance the stemness of ISCs and intestinal regeneration. These findings reveal that *Snord15b*‐Ilf2 association mediates alternative splicing of *Btrc* short isoform to inhibit β‐catenin ubiquitination for ISC stemness maintenance.

## Graphical abstract

In intestinal stem cells (ISCs), *Snord15b* associates with Ilf2 to recruit splicing factors for alternative splicing of *Btrc* mRNA, leading to the generation of short Btrc. This short Btrc fails to form a functional E3 ubiquitin ligase complex for β‐catenin ubiquitination and degradation. As a result, more stable β‐catenin translocates into the nucleus to activate the Wnt/β‐catenin pathway, facilitating ISC stemness maintenance.

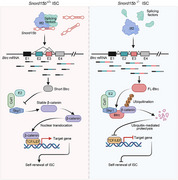

## 1 Introduction

The intestinal epithelium undergoes rapid cellular turnover and regeneration every 3—7 days, supported by the intestinal stem cells (ISCs) localized in intestinal crypts. ISCs exhibit sustained self‐renewal and differentiation capabilities, producing five major differentiated epithelial cells, including enterocyte, goblet, enteroendocrine, Paneth, and tuft cell.^[^ ^1^, ^2^ ^]^ Lineage‐tracing studies have identified distinct populations of ISCs: rapidly dividing Lgr5^+^ ISCs (called crypt base columnar cells) resided at the crypt base,^[^ ^3^ ^]^ a quiescent population of cells identified by the marker Bmi1 at the +4 cell position.^[^ ^4^, ^5^ ^]^ Of note, newly defined Fgfbp1^+^ ISCs are located at the upper crypt, and isthmus progenitors express Lgr4.^[^ ^6^, ^7^ ^]^ ISCs play a critical role in intestinal development, homeostasis maintenance, and injury repair. Furthermore, an imbalance in the homeostasis of ISCs leads to inflammatory bowel disease and colorectal cancer. Elucidating the mechanisms of ISC stemness maintenance has substantial clinical significance for the treatment of intestinal diseases.^[^ ^8^, ^9^ ^]^

ISCs are regulated by a complex signaling network within their specialized niche, including Wnt, Notch, BMP, EGF, and Hedgehog signaling.^[^ ^10^ ^]^ The coordinated regulation of various signaling networks in the ISC niche is important for maintaining the self‐renewal and differentiation of ISCs. For instance, instability of β‐catenin and inactivation of Wnt/β‐catenin signaling impairs ISC self‐renewal.^[^ ^11^, ^12^ ^]^ Although the signaling pathways for the homeostasis of ISCs have been extensively investigated, the mechanisms underlying the self‐renewal of ISCs are still elusive.

Small nucleolar RNAs (snoRNAs) are a class of small non‐coding RNAs found abundantly in the nucleolus of eukaryotic cells, ranging from 60 to 300 nucleotides in length.^[^ ^13^ ^]^ SnoRNAs are primarily classified into two main types based on their conserved sequence structures: C/D box and H/ACA box snoRNAs, separately mediating the 2′‐O‐methylation and pseudouridylation of ribosomal RNA (rRNA) via binding to ribonucleoproteins (RNPs) to form functional snoRNP complexes, which play critical roles in rRNA processing and ribosome assembly.^[^ ^14^ ^]^ However, emerging evidence indicates that some orphan snoRNAs exert regulatory effects through non‐canonical mechanisms. For instance, *SNORD50A/B* are specifically located in the cytoplasm and directly interact with K‐Ras, leading to its degradation and thereby inhibiting the progression of human cancers.^[^ ^15^ ^]^ *SNORA73* targets mRNAs that encode secretory proteins and membrane proteins, acting as a molecular ternary glue to enhance the association of target mRNAs with the signal recognition particle for protein secretion.^[^ ^16^ ^]^ Adipocyte‐expressed *SNORD46* competitively binds to IL‐15, inhibiting IL‐15‐dependent autophagy in NK cells, thus leading to impaired anti‐tumor immunity of NK cells under obesity conditions.^[^ ^17^ ^]^ SnoRNA HBII‐52 regulates alternative splicing of serotonin receptor 2c (Htr2c) to promote expression of functional receptor.^[^ ^18^ ^]^ Here, we identified a conserved snoRNA, *Snord15b* (originated from the *Rps3* gene transcript, NCBI ID: 449 631) that was highly expressed in ISCs. We showed that *Snord15b* knockout impaired ISC self‐renewal and epithelial regeneration. *Snord15b*‐Ilf2 association mediated alternative splicing of *Btrc* to form a short isoform of Btrc, suppressing β‐catenin ubiquitination to activate Wnt/β‐catenin signaling for ISC self‐renewal maintenance.

## 2 Results

### 2.1 *Snord15b* is Highly Expressed in Lgr5^+^ ISCs

**Figure 1:**
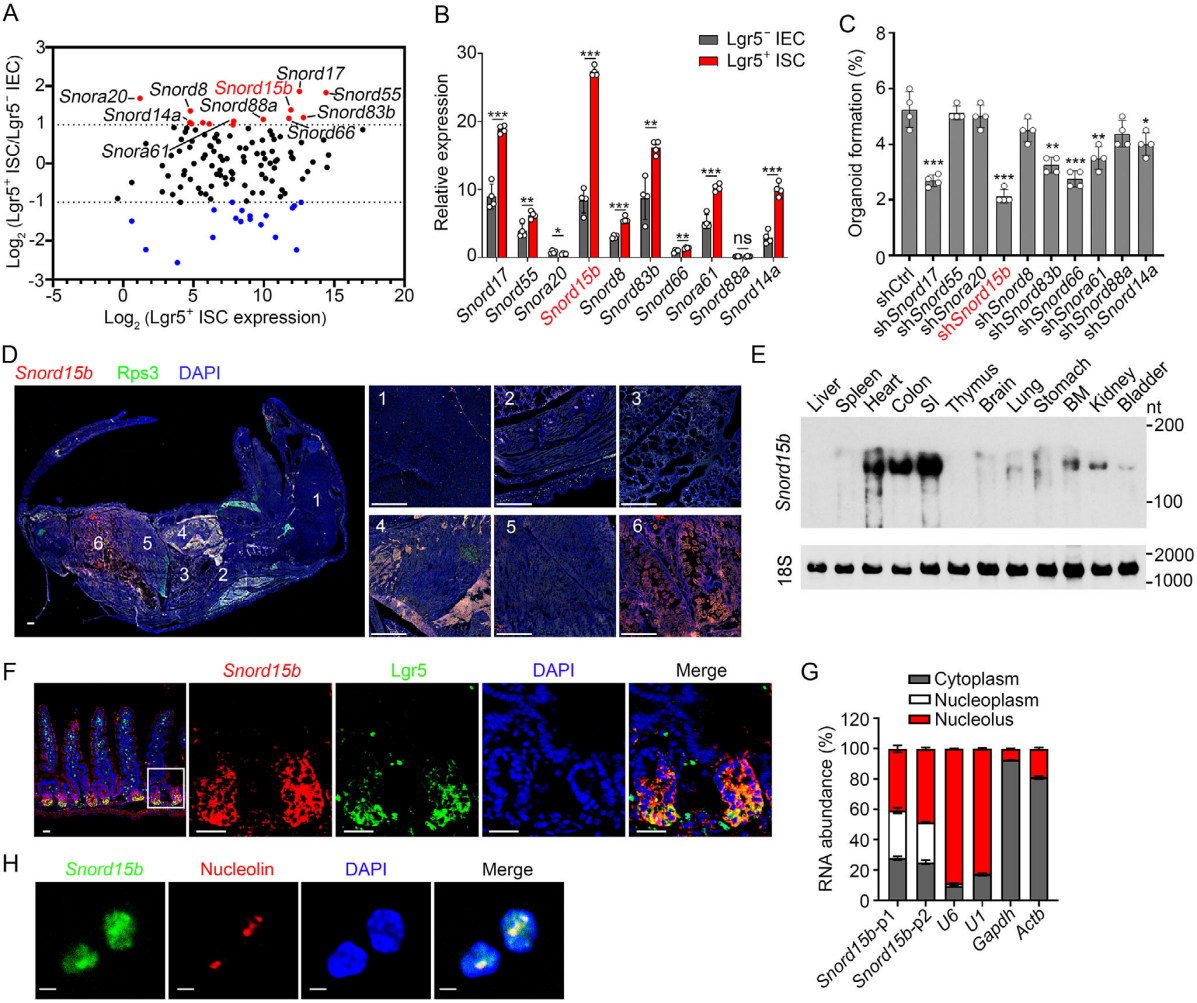
*Snord15b* is highly expressed in ISCs. A) Lgr5^+^ and Lgr5^−^ cells were sorted from intestinal crypts of *Lgr5* ^GFP^ mice and performed snoRNA sequencing. Data are shown as a scatter plot of snoRNA in Lgr5^+^ and Lgr5^−^ cells, and the top 10 upregulated snoRNAs with high abundance in Lgr5^+^ cells are marked. B) Relative mRNA levels of marked snoRNAs above in Lgr5^+^ and Lgr5^−^ cells were analyzed by qRT‐PCR. *n* = 4 independent experiments. Data of qRT‐PCR were normalized to endogenous U6 unless noted in this study. C) Intestinal crypts were isolated from C57BL/6 mice for organoid culture. The top 10 upregulated snoRNAs marked above were silenced in ISCs via shRNA, followed by organoid formation. Organoid formation ratios per well were analyzed. *n* = 4 wells per group. D) One‐week‐old mice were euthanized for longitudinal sections. A global view of the section is shown in the left panel, and the indicated tissues are shown in the right. 1, brain; 2, bone marrow; 3, lung; 4, heart; 5, liver; 6, intestine. Scale bars, 500 µm. E) Northern blotting analysis of *Snord15b* in different tissues of mice. 18S rRNA was used as a loading control. F) *Snord15b* was visualized by FISH in small intestine tissues. Scale bars, 30 µm. G) Distribution of *Snord15b* in mouse ISCs was determined by qRT‐PCR. U1 and U6 served as positive controls for nuclear location. *Gapdh* and *Actb* served as cytoplasmic RNA markers. *n* = 3 independent experiments. H) RNA FISH of *Snord15b* in ISCs. Scale bars, 4 µm. Data are shown as the means ± SD. Statistical analysis was performed using one‐way ANOVA with Tukey's multiple comparisons testing (C); comparisons across groups for a single variable were performed via multiple unpaired Student's *t*‐tests with Holm–Šidák correction (B). *P < 0.05, **P < 0.01, ***P < 0.001, ns, not significant.

To explore the role of snoRNAs in the modulation of ISCs, we isolated Lgr5^+^ ISCs and Lgr5^−^ intestinal epithelial cells (IECs) from small intestines of *Lgr5* ^GFP^ mice and human intestinal samples, followed by snoRNA sequencing (**Figure** **1**A; Figure S1A, Supporting Information). We then selected the top 10 upregulated snoRNAs with high abundance in mouse Lgr5^+^ ISCs and validated their higher expression through qRT‐PCR (Figure 1B). Subsequently, we knocked down these 10 snoRNAs individually via short hairpin RNA (shRNA) in ISCs and assessed their impact on ISC stemness through organoid formation assays. We found that *Snord15b* knockdown most significantly inhibited organoid formation (Figure 1C; Figure S1B,C, Supporting Information). Thus, we focused on *Snord15b* in the stemness maintenance of intestinal ISCs.

*Snord15b* is located in the intronic region between the fifth and sixth exons of the *Rps3* gene locus on mouse chromosome 7, with a length of 145 nt. *Snord15b* is conserved across various species (Figure S1D,E, Supporting Information). According to the NCBI and snoRNA orthological gene database (snOPY) databases, the *Rps3* gene harbors another homologous transcript *Snord15a*, which exhibits ≈60% sequence similarity to *Snord15b*. However, *Snord15b* showed substantial abundance in intestinal crypts and Lgr5^+^ ISCs compared to *Snord15a* (Figure S1F,G, Supporting Information). In addition, we observed that expression levels of *Snord15b* in Lgr5^+^ ISCs with *Snord15b* depletion were lower than that in Lgr5^−^ IECs (Figure S1H, Supporting Information). *Snord15b* was expressed in several tissues, including heart, colon, small intestine, kidney, and bone marrow. However, it was most substantially expressed in the small intestines (Figure 1D,E). *Snord15b* was mainly highly expressed in crypt epithelium compared with villus epithelium of the small intestine (Figure 1F). Furthermore, *Snord15b* was specifically enriched at the tips of budding structures in intestinal organoids, which was consistent with the crypt regions of the small intestines (Figure S1I, Supporting Information). Subcellular fractionation demonstrated that *Snord15b* was distributed in cytoplasm (≈30%), nucleoplasm (≈30%), and nucleolus (≈40%) within ISCs (Figure 1G; Figure S1J, Supporting Information), which was further validated by RNA‐fluorescence in situ hybridization (RNA‐FISH) assays (Figure 1H). *SNORD15B* was also highly expressed in human ISCs derived from human intestine tissue samples (Figure S1K, Supporting Information). Collectively, these data indicate that *Snord15b* is highly expressed in mouse ISCs.

### 2.2 *Snord15b* Knockout Impairs Stemness of ISCs and Intestinal Regeneration

**Figure 2:**
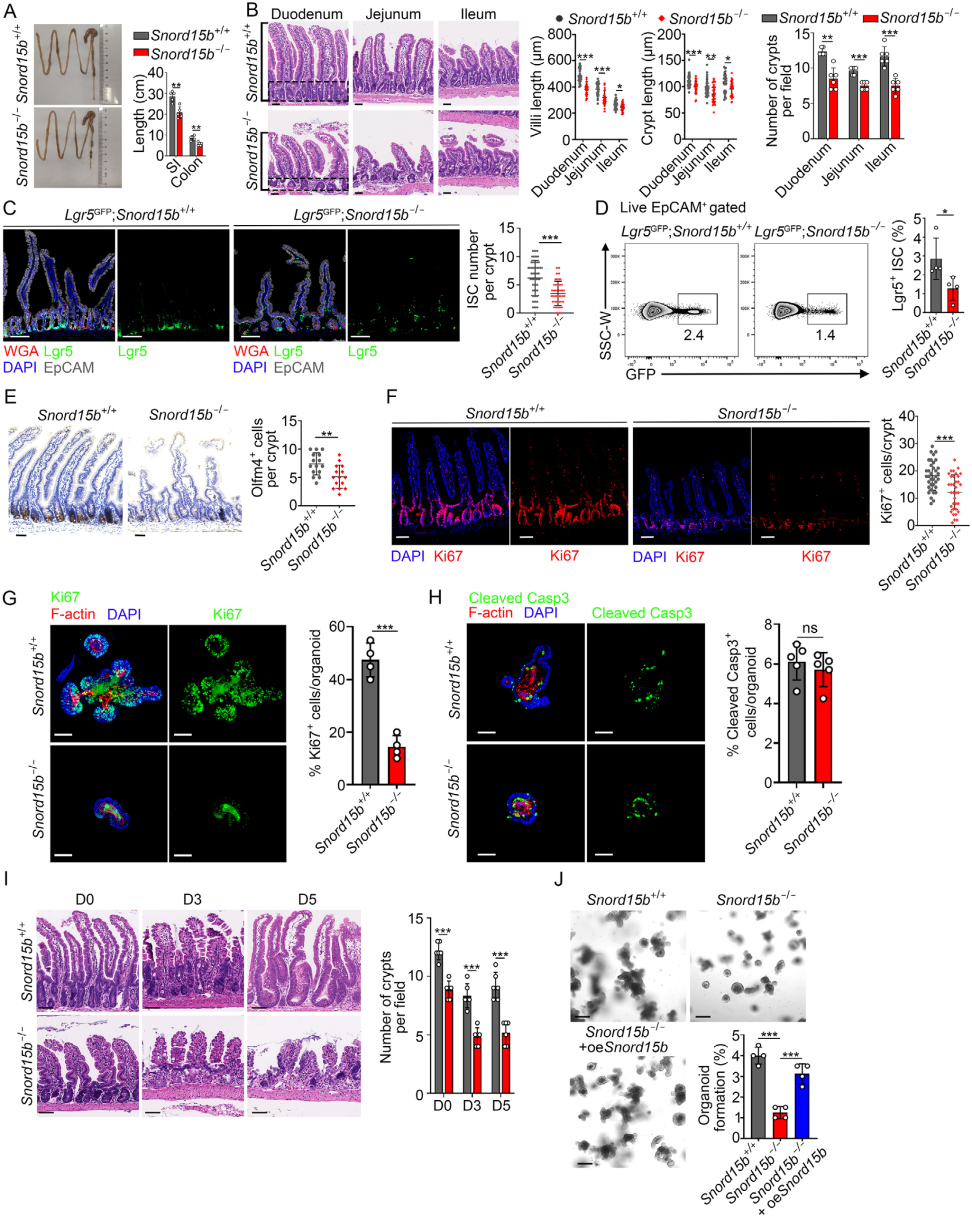
*Snord15b* knockout impairs self‐renewal of ISCs. A) Small intestines and colon images of *Snord15b* ^+/+^ and *Snord15b* ^−/−^ mice. The length of the small intestines and the colon was calculated. *n* = 5 mice for each group. B) Representative H&E staining images of three small intestine regions: duodenum, jejunum, and ileum from *Snord15b* ^+/+^ and *Snord15b* ^−/−^ mice. Scale bars, 50 µm. Length of villi and crypts, as well as crypt numbers per field are shown in the right panel. *n* = 30 villi or crypts for length calculation. *n* = 6 fields for crypt number calculation. C) *Lgr5* ^GFP^ mice were crossed with *Snord15b* ^−/−^ mice for Lgr5 observation. Scale bars, 100 µm. Statistical ISC numbers per crypt are shown in the right panel. *n *= 60 crypts for each group. D) FACS analysis of GFP^+^ ISCs in *Lgr5* ^GFP^; *Snord15b* ^+/+^ and *Lgr5* ^GFP^; *Snord15b* ^−/−^ mice. Ratios of GFP^+^ ISCs in live EpCAM^+^ epithelial cells are shown in the right panel. *n* = 4 mice for each group. E) Small intestines of *Snord15b* ^+/+^ and *Snord15b* ^−/−^ mice were stained with Olfm4 antibody. Immunohistochemical staining images of the jejunum were shown as a representative. Statistical Olfm4^+^ cell numbers per crypt are shown in the right panel. *n* = 15 crypts for each group. F) Immunofluorescence staining of Ki67^+^ cells in *Snord15b* ^+/+^ and *Snord15b* ^−/−^ mouse intestine sections. Scale bars, 70 µm. Numbers of Ki67^+^ cells per crypt are shown in the right panels. *n* = 35 crypts for each group. G) Immunofluorescence staining of Ki67^+^ cells in *Snord15b* ^+/+^ and *Snord15b* ^−/−^ intestinal organoids. Scale bars, 50 µm. Ratios of Ki67^+^ cells per organoid are shown in the right panel. *n* = 4 organoids for each group. H) Immunofluorescence staining of apoptotic cells expressing cleaved caspase3 (C‐Caspase3) in *Snord15b* ^+/+^ and *Snord15b* ^−/−^ intestinal organoids. Scale bars, 50 µm. Ratios of C‐Caspase3^+^ cells per organoid are shown in the right panel. *n *= 5 organoids for each group. I) *Snord15b* ^+/+^ and *Snord15b* ^−/−^ mice were treated with 8 Gy of radiation, and small intestines were isolated for H&E staining at days 0, 3, 5. H&E staining results of the jejunum were presented as a representative. Crypt numbers per field are shown in the right panel. Scale bars, 70 µm. *n* = 5 fields for each group. J) Organoid formation was conducted in *Snord15b* ^+/+^, *Snord15b* ^−/−^ and *Snord15b* ^−/−^ + oe*Snord15b* ISCs. Lentivirus was used for the overexpression of *Snord15b*. Scale bars, 200 µm. Ratios of organoid formation per well were calculated and shown in the lower panel. *n* = 4 wells for each group. Data are shown as the means ± SD. Statistical analysis was performed using unpaired two‐tailed Student's *t*‐tests (C–H) and one‐way ANOVA with Tukey's multiple comparisons testing (J); comparisons across groups for a single variable were performed via multiple unpaired Student's *t‐*tests with Holm–Šidák correction (A,B,I). *P < 0.05, **P < 0.01, ***P < 0.001, ns, not significant.

To determine the physiological role of *Snord15b*, we generated *Snord15b* knockout (*Snord15b* ^−/−^) mice using CRISPR/Cas9 technology (Figure S2A, Supporting Information). *Snord15b* was completely deleted in ISCs from *Snord15b* ^−/−^ mice through validation by genotyping, qRT‐PCR, and Northern blotting (Figure S2B–D, Supporting Information). Of note, *Snord15b* deletion did not affect expression of *Snord15a* and its host gene *Rps3* (Figure S2E–G, Supporting Information). Importantly, the small intestines and colons of *Snord15b* ^−/−^ mice were much shorter in length than those of *Snord15b* ^+/+^ mice (**Figure** **2**A). Furthermore, *Snord15b* ^−/−^ mice exhibited markedly shorter crypts and villi, along with reduced numbers of crypts across all three regions of small intestine (duodenum, jejunum, and ileum) (Figure 2B). Additionally, *Snord15b* ^−/−^ mice showed decreased Lgr5^+^ ISCs in small intestines and colons (Figure 2C,D; Figure S2H, Supporting Information). Reduced ISCs in *Snord15b* ^−/−^ mice were also validated by other ISC markers, such as Olfm4 (Figure 2E). In parallel, Ki67 staining exhibited decreased proliferation of *Snord15b* ^−/−^ intestinal and colonic ISCs (Figure 2F,G; Figure S2I, Supporting Information). However, cleaved caspase3 and TUNEL staining showed no overt difference between *Snord15b* ^+/+^ and *Snord15b* ^−/−^ intestinal ISCs (Figure 2H; Figure S2J, Supporting Information).

Considering involvement of Lgr5^+^ ISCs in radiation‐induced intestinal regeneration,^[^ ^19^ ^]^ we subsequently assessed renewal of intestinal epithelia in *Snord15b* ^−/−^ mice. Following radiation‐induced gut epithelial injury (8 Gy), *Snord15b* ^−/−^ mice exhibited impaired regeneration of intestinal epithelia compared to their *Snord15b^+/+^* littermates (Figure 2I). Meanwhile, we noticed that the organoid formation capacity of ISCs derived from *Snord15b* ^−/−^ mice was reduced. While overexpression of *Snord15b* in *Snord15b* ^−/−^ ISCs effectively restored their ability to form organoids, but *Snord15a* overexpression in *Snord15b* ^−/−^ ISCs did not achieve the same restoration (Figure 2J; Figure S2K–M, Supporting Information). Altogether, *Snord15b* is required for the intestinal epithelial self‐renewal.

### 2.3 Snord15b Associates with Ilf2 in ISCs

We wanted to explore the molecular mechanism by which *Snord15b* contributes to the maintenance of ISCs' stemness. Given that our results indicated a nucleolar distribution of *Snord15b*, we first examined whether *Snord15b* deletion affected the nucleolus of ISCs. We observed that *Snord15b* knockout did not change the number of nucleoli in ISCs (Figure S3A, Supporting Information). snOPY database predicted that *Snord15b* mediated the 2′‐O‐methylation of 28S rRNA at A3441 position (Figure S3B, Supporting Information). We analyzed 2′‐O‐methylation of 28S rRNA at A3441 in *Snord15b* ^+/+^ and *Snord15b* ^−/−^ ISCs using a reverse transcription approach with limiting dNTP concentrations^[^ ^20^ ^]^ (Figure S3B, Supporting Information). Under high dNTP concentrations, similar amplification levels were observed between *Snord15b* ^+/+^ and *Snord15b* ^−/−^ ISCs. In contrast, at low dNTP concentrations, amplification levels increased, indicating that *Snord15b* deletion reduced 2′‐O‐methylation at 28S‐A3441 (Figure S3C, Supporting Information). Furthermore, the reduction in 2′‐O‐methylation at 28S‐A3441 caused by *Snord15b* knockout could be rescued by overexpression of *Snord15b* (oe*Snord15b*), but not by mutated *Snord15b* (oe*Snord15b* ^mut^), which carried a mutation in the region binding with 28S rRNA (Figure S3D, Supporting Information). However, the loss of 2′‐O‐methylation at 28S‐A3441 did not affect organoid formation of ISCs (Figure S3E, Supporting Information). Collectively, these findings suggest that *Snord15b* is involved in the regulation of ISC stemness in a non‐classical pathway.

**Figure 3:**
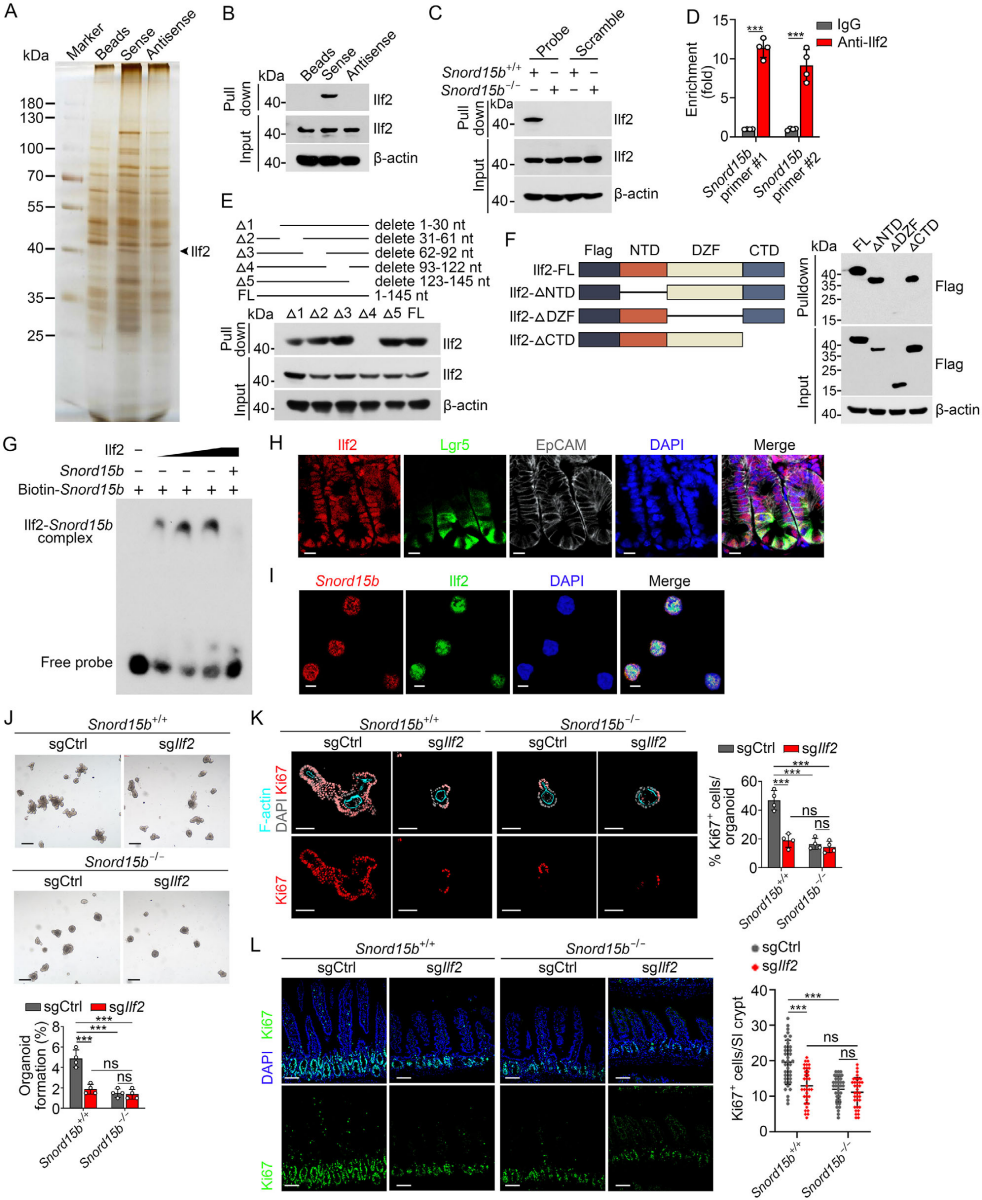
*Snord15b* interacts with Ilf2 in ISCs. A) Intestinal crypts from WT mice were lysed and incubated with biotin‐labeled sense (*Snord15b* transcript), antisense, and Sepharose 4B beads control. Eluted fractions were resolved by SDS–PAGE followed by silver staining and mass spectrometry. The black arrowhead indicates the Ilf2 band. B) Interaction between *Snord15b* and Ilf2 was confirmed by Western blotting. C) Interaction between *Snord15b* and Ilf2 was validated by RAP assay. D) RIP assay was conducted using anti‐Ilf2 antibody or IgG in crypts lysates. *n* = 4 independent experiments. E) Schematic diagram of truncated *Snord15b*. Truncated *Snord15b* was biotin‐labeled and incubated with intestinal crypts lysates, followed by RNA pulldown assay and Western blot. F) Domain mapping of *Snord15b*‐binding domains of Ilf2 protein. Different truncated Ilf2 were incubated with *Snord15b*, followed by RNA pulldown assay and Western blotting. G) Recombinant Ilf2 and biotin‐labeled *Snord15b* were incubated for EMSA. H) Colocalization of Ilf2^+^ and Lgr5^+^ cells in intestinal crypts was visualized by immunofluorescence staining. Scale bars, 10 µm. I) Colocalization of *Snord15b* and Ilf2 in Lgr5^+^ ISCs was visualized by RNA FISH. Scale bars, 5 µm. J) Knockout of *Ilf2* was conducted in *Snord15b* ^+/+^ and *Snord15b* ^−/−^ organoids using the CRISPR/Cas9 system, followed by an organoid formation assay. Scale bars, 200 µm. Organoid formation ratios per well are shown. *n* = 4 wells for each group. K) Immunofluorescence staining of Ki67 in *Ilf2* KO and control organoids from *Snord15b* ^+/+^ and *Snord15b* ^−/−^ mice. Scale bars, 70 µm. Ratios of Ki67^+^ cells per organoid are shown in the right panel. *n* = 4 organoids for each group. L) Tamoxifen‐pretreated *Lgr5* ^GFP‐CreERT2^; *Rosa26* ^lsl‐Cas9^ mice were infected with lentivirus carrying sgRNAs for *Ilf2*, and euthanized two weeks later. Small intestines from indicated mice were conducted for immunofluorescence. Scale bars, 80 µm. Numbers of Ki67^+^ cells per crypt were calculated. *n* = 35 crypts for each group. Data are shown as the means ± SD. Comparisons across groups for a single variable were performed via multiple unpaired Student's *t*‐tests with Holm–Šidák correction (D,J–L). ***P < 0.001, ns, not significant.

We next performed an RNA pulldown assay to identify associated protein candidates of *Snord15b*. Interleukin enhancer‐binding factor 2 (Ilf2) was identified as a candidate protein associated with *Snord15b* (**Figure** **3**A; Figure S4A, Supporting Information). The interaction between *Snord15b* and Ilf2 was further validated using Western blotting (Figure 3B). Notably, mutating the 28S‐A3441 binding region of *Snord15b* did not affect its binding affinity for Ilf2 (Figure S4B, Supporting Information). As expected, the enrichment of Ilf2 by *Snord15b* was remarkably diminished in *Snord15b* ^−/−^ mice (Figure 3C). Additional RNA immunoprecipitation (RIP) assays also confirmed that *Snord15b* directly bound Ilf2 (Figure 3D). Furthermore, 3D structure prediction via HDOCK Web Servers indicated an interaction between *Snord15b* and Ilf2 (Figure S4C, Supporting Information). RNA truncation assay revealed that the 93–122 nt fragment of *Snord15b* was required for its binding to Ilf2 (Figure 3E). Through domain mapping assays, we determined that the DZF domain of Ilf2 was required for binding with *Snord15b* (Figure 3F). Consistently, RNA electrophoretic mobility shift assays (EMSA) showed a strong interaction between *Snord15b* and endogenous Ilf2 (Figure 3G). Immunofluorescence staining revealed that Ilf2 was expressed in Lgr5^+^ cells (Figure 3H). The colocalization of *Snord15b* with Ilf2 in ISCs was further validated by RNA‐FISH assays (Figure 3I). To further examine the role of Ilf2 in the regulation of ISCs, we thus generated *Ilf2*‐deficient mice (*Snord15b* ^+/+^;sg*Ilf2* and *Snord15b* ^−/−^;sg*Ilf2*) using CRISPR/Cas9‐mediated genome editing. We observed that knockout of *Ilf2*, as well as double knockout of *Snord15b* with *Ilf2*, remarkably impaired organoid formation capacity of mouse ISCs, which was consistent with the finding in *Snord15b* ^−/−^ mice (Figure 3J). Subsequently, we found that *Ilf2* deficiency decreased the number of proliferating cells in the small intestines (Figure 3K,L). In parallel, we observed that deletion of *SNORD15B* or *ILF2* in human colonic crypts also suppressed organoid formation (Figure S4D,E, Supporting Information). It has been reported that Lgr5^+^ stem cells are the source of cancer stem cells for small intestinal adenoma and colorectal cancer (CRC).^[^ ^21^, ^22^ ^]^ Thus, we determined expression levels of *SNORD15B* in CRC samples and found that *SNORD15B* was highly expressed in colon tumors (Figure S4F,G, Supporting Information), suggesting *SNORD15B* could be involved in the intestinal tumorigenesis. Taken together, these results indicate that *Snord15b* regulates the stemness of ISCs through direct interaction with Ilf2.

### 2.4 *Snord15b*‐Ilf2 Association Mediates Alternative Splicing of *Btrc*

**Figure 4:**
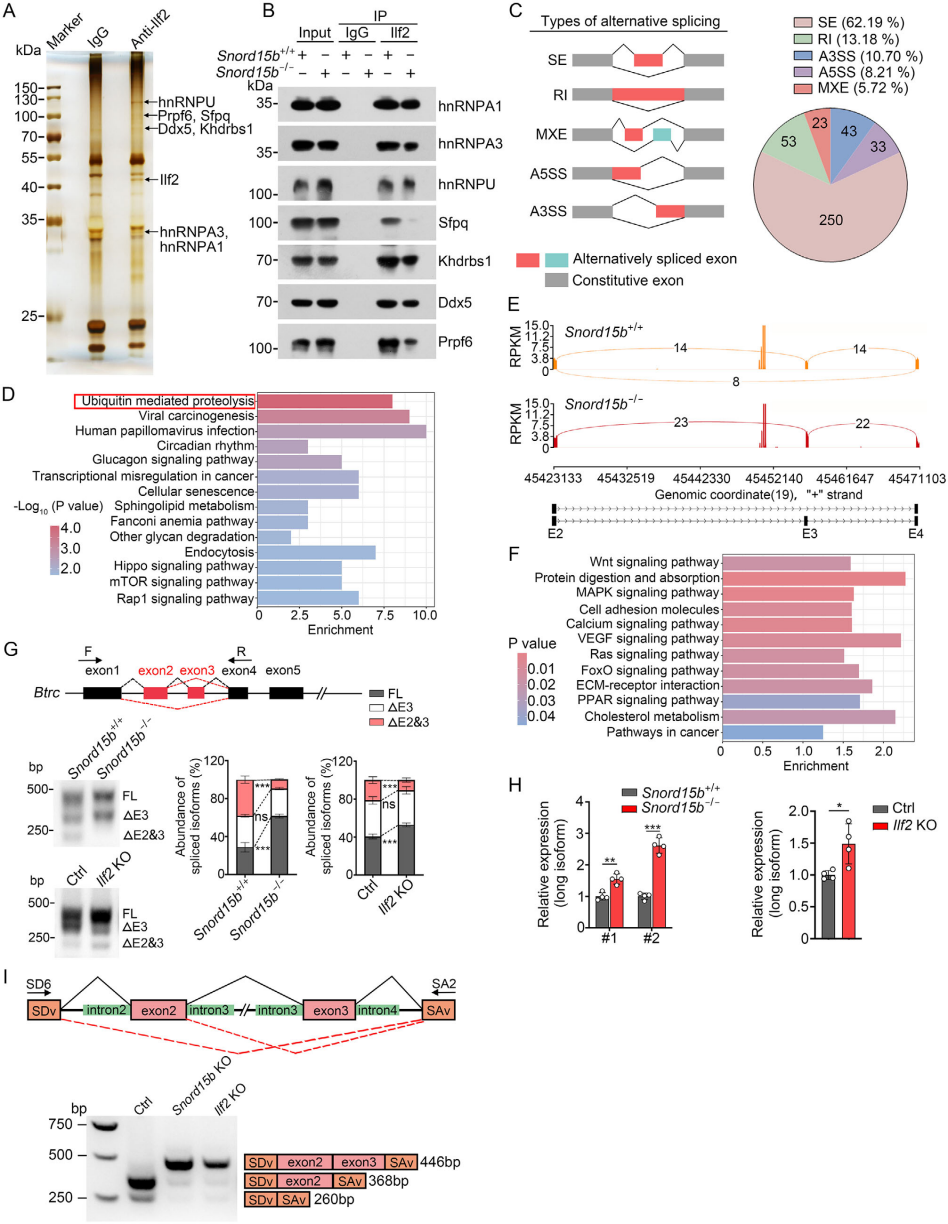
*Snord15b‐*Ilf2 association regulates alternative splicing of *Btrc* mRNA. A) Co‐IP was performed on Ilf2. Ilf2 antibody or IgG was incubated with crypt lysates, then Protein A/G was used for Ilf2 binding protein enrichment. Enriched components were separated by SDS‐PAGE followed by silver staining and mass spectrometry. Black arrows indicate Ilf2‐binding splicing factors. B) Interaction between Ilf2 and splicing factors in *Snord15b* ^+/+^ and *Snord15b* ^−/−^ mice was confirmed by Western blotting. C) Schematic diagram of alternative splicing (AS) types. The pie chart depicts proportions of different types of AS events in RNA‐seq data from intestinal ISCs of *Snord15b* KO mice and littermate controls. D) KEGG analysis based on differentially expressed AS genes between *Snord15b* ^+/+^ and *Snord15b* ^−/−^ ISCs. E) Representative Sashimi plots depicting alternative splicing patterns of *Btrc* mRNA in *Snord15b* ^+/+^ and *Snord15b* ^−/−^ ISCs. Horizontal and vertical axes represent per‐base expression and genomic coordinates, respectively. mRNA isoforms quantified were given at the bottom; black boxes and lines with arrow heads represent exons and introns, respectively. F) KEGG analysis of total differentially expressed genes between *Snord15b* ^+/+^ and *Snord15b* ^−/−^ ISCs. G) Verification of Btrc mRNA isoforms in *Snord15b* KO, *Ilf2* KO, and their corresponding organoids via semi‐quantitative PCR. An abundance of different isoforms was calculated and shown in the right panel. *n* = 4 independent experiments. H) Btrc long isoform was detected in *Snord15b* KO, *Ilf2* KO, and their corresponding control intestinal organoids. *n* = 4 independent experiments. I) Analysis of splicing of *Btrc* minigene by semi‐quantitative PCR. Data are shown as the means ± SD. Comparisons across groups for a single variable were performed via multiple unpaired Student's *t*‐tests with Holm–Šidák correction (G,H). *P < 0.05, **P < 0.01, ***P < 0.001, ns, not significant.

To determine how the association of *Snord15b* with Ilf2 modulated ISC stemness, we performed a co‐immunoprecipitation (co‐IP) assay to identify potential interacting proteins of Ilf2 from crypt lysates (**Figure** **4**A). Through mass spectrometry, several proteins associated with Ilf2 were defined to be implicated in mRNA alternative splicing (Figure S5A, Supporting Information). In addition, the interactions between Ilf2 and several splicing factors were confirmed by Western blotting. Of note, *Snord15b* deletion abrogated the association of Ilf2 with splicing factors (Figure 4B; Figure S4H, Supporting Information). Alternative splicing (AS) is a pivotal regulatory mechanism at the RNA level, and aberrant AS disrupts ISC homeostasis.^[^ ^23^, ^24^ ^]^ To determine whether dysregulated AS events contributed to ISC stemness regulation, we conducted RNA sequencing (RNA‐seq) on Lgr5^+^ ISCs isolated from *Snord15b* ^+/+^ and *Snord15b* ^−/−^ mice, followed by analysis of AS events. 402 genes exhibited differential AS events (FDR < 0.05) between *Snord15b* ^+/+^ and *Snord15b* ^−/−^ ISCs, including five distinct types of alternative splicing (Figure 4C). Further Kyoto Encyclopedia of Genes and Genomes (KEGG) pathway analysis on these 402 aberrantly spliced genes (UASGs) showed that the most significant enrichment was observed in the ubiquitin‐mediated proteolysis pathway (Figure 4D). The aberrant AS of five representative genes in this pathway, caused by either *Snord15b* or *Ilf2* knockout, was further validated via semi‐quantitative PCR (Figure 4E; Figure S5B,C, Supporting Information).

To further determine how the AS of genes involved in protein stability regulation affected ISC stemness maintenance, we conducted another KEGG pathway analysis on total differentially expressed genes between *Snord15b* ^+/+^ and *Snord15b* ^−/−^ ISCs. We found that downregulated genes in *Snord15b*‐deficient ISCs were predominantly enriched in the Wnt signaling pathway (Figure 4F). Subsequent qRT‐PCR revealed that *Snord15b* knockout reduced the expression of target genes downstream of the Wnt signaling pathway (Figure S5D, Supporting Information). Among the aforementioned five UASGs related to ubiquitin‐mediated proteolysis, aberrant AS of *BTRC* affecting Wnt signaling was reported in the human brain,^[^ ^25^ ^]^ but the functional implications of *Snord15b*‐Ilf2‐mediated *Btrc* splicing in ISCs remain unclear. Through semi‐quantitative PCR and qRT‐PCR using isoform‐specific primers, we validated that knockout of either *Snord15b* or *Ilf2* disrupted skipping of *Btrc* exons 2 and 3, leading to increased abundance of full‐length (FL) *Btrc* (Figure 4G,H; Figure S6A, Supporting Information). Analysis of AS events throughout the *Btrc* full‐length CDS region further revealed that divergent AS due to *Snord15b* or *Ilf2* deficiency did not occur in other exons except for exons 2 and 3 (Figure S6B, Supporting Information). In addition, alternative splicing of exons 2 and 3 had no impact on the translation of *Btrc*. The spliced isoforms exhibited no discernible band shift in Western blotting, attributable to a minimal molecular weight difference (6.8 kDa) between the long and short isoforms (Figure S6C, Supporting Information). Through a minigene assay, we further confirmed that depletion of *Snord15b* or *Ilf2* remarkably enhanced the generation of FL‐*Btrc* (Figure 4I). Based on our previous results (Figure S4H, Supporting Information), we found that *Snord15b* deletion most remarkably affected the binding between Ilf2 and the splicing factor Sfpq. We then depleted *Sfpq* in intestinal organoids and observed that *Sfpq* deficiency influenced alternative splicing of *Btrc*, leading to increased generation of FL‐*Btrc* (Figure S6D, Supporting Information). Taken together, these findings indicate that *Snord15b* modulates the alternative splicing of genes critical for ubiquitin‐mediated proteolysis by mediating the interaction of Ilf2 with specific splicing factors.

### 2.5 *Snord15b*‐Ilf2 Association Promotes *Btrc* Exon Skipping to Inhibit Ubiquitination and Degradation of β‐Catenin

**Figure 5:**
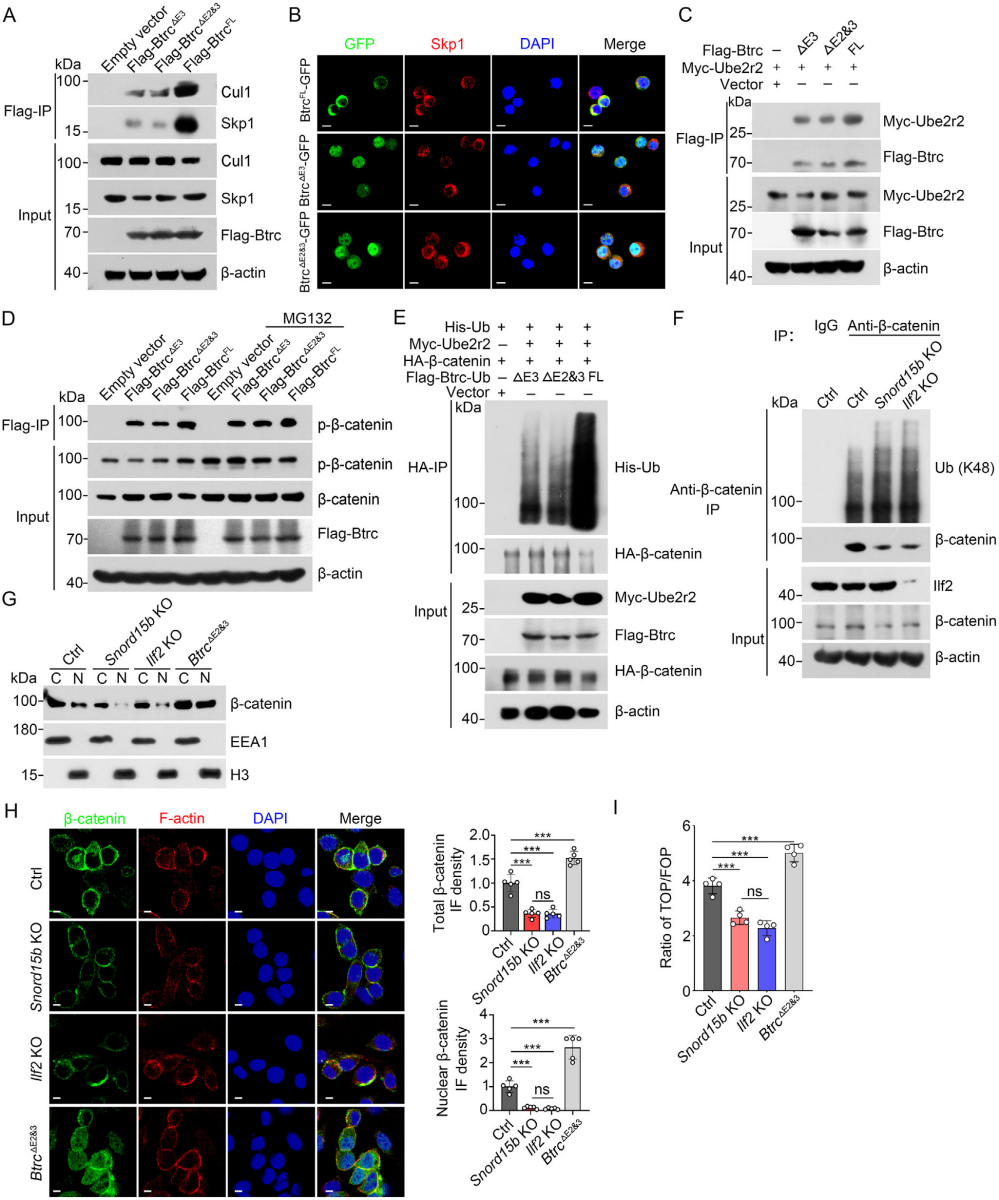
Short isoforms of Btrc attenuate β‐catenin ubiquitination to inhibit its degradation. A) Flag‐tagged Btrc isoforms (△E3, △E2&3, FL) were transfected into HEK293T cells for 48 h. Cell lysates were incubated with anti‐Flag antibody for an immunoprecipitation assay, followed by Western blotting with anti‐Flag, anti‐Skp1, and anti‐Cul1 antibodies. B) GFP‐tagged Btrc isoforms (△E3, △E2&3, FL) were transfected into MC38 cells for 48 h. Location of different Btrc isoforms and their interaction with Skp1 were visualized by immunofluorescence staining. Scale bars, 10 µm. C) Myc‐tagged Ube2r2 and Flag‐tagged Btrc isoforms (△E3, △E2&3, FL) were cotransfected into HEK293T cells for 48 h. Cell lysates were incubated with anti‐Flag antibody for an immunoprecipitation assay, followed by Western blotting with anti‐Myc and anti‐Flag antibodies. D) Flag‐tagged Btrc isoforms (△E3, △E2&3, FL) were transfected into HEK293T cells for 36 h. Then cells were treated with MG132 (10 µm) for 12 h. Cell lysates were incubated with anti‐Flag antibody for an immunoprecipitation assay, followed by Western blotting with anti‐phospho‐β‐catenin antibodies. E) Myc‐tagged Ube2r2, Flag‐tagged Btrc isoforms (△E3, △E2&3, FL), HA‐tagged β‐catenin, and His‐tagged WT ubiquitin were cotransfected into HEK293T cells for 48 h followed by Western blotting. F) Lysates from WT, *Snord15b* KO, and *Ilf2* KO intestinal organoids were incubated with anti‐β‐catenin antibody for immunoprecipitation, followed by Western blotting with K48‐linked specific ubiquitination antibody. G) Western blotting analysis of β‐catenin in nuclear and cytoplasmic fractionated extracts from WT, *Snord15b* KO, *Ilf2* KO, and *Btrc* (△E2&3) MC38 cells. EEA1 and H3 are protein markers for the cytoplasm and nucleus, respectively. H) β‐catenin distribution in nuclei and cytoplasm of WT, *Snord15b* KO, *Ilf2* KO, and *Btrc* (△E2&3) MC38 cells was visualized via Immunofluorescence staining. Scale bars, 10 µm. Fluorescence intensity of total and nuclear β‐catenin per field was calculated and compared with the WT group. *n* = 5 fields for each group. I) WT, *Snord15b* KO, *Ilf2* KO, and *Btrc* (△E2&3) MC38 cells were transfected with TOP‐flash (TOP) or FOP‐flash (FOP) luciferase‐reporter vectors. After 24 h transfection, relative luciferase activity was calculated, and the TOPflash/FOPflash ratio was shown. *n *= 4 independent experiments. Data are shown as the means ± SD. Statistical analysis was performed using one‐way ANOVA with Tukey's multiple comparisons testing (H,I). *P < 0.05, **P < 0.01, ***P < 0.001, ns, not significant.

Btrc is a key component of the SCF (Skp1‐Cul1‐Btrc) E3 ubiquitin ligase complex, which facilitates ubiquitination and subsequent degradation of target proteins. One of the well‐known substrates of this complex is β‐catenin, whose degradation is regulated by SCF‐mediated ubiquitination^[^ ^25^ ^]^ (Figure S7A, Supporting Information). To determine the impact of the short isoform of Btrc on the stability of β‐catenin, we performed a co‐IP assay to evaluate the interaction between different Btrc isoforms and its partners, Skp1 and Cul1. We found that short isoforms of Btrc exhibited reduced binding affinity to SCF proteins (**Figure** **5**A; Figure S7B, Supporting Information). Consistently, reduced colocalization of short Btrc isoforms and Skp1 was observed in the cytoplasm (Figure 5B). It has been reported that the ubiquitin‐conjugating enzyme Ube2r2 mediates SCF‐catalyzed substrate polyubiquitination.^[^ ^26^ ^]^ We observed that the interaction between short Btrc and Ube2r2 was weakened (Figure 5C; Figure S7C, Supporting Information). Deletion of *Btrc* exons 2 and 3 reduced its binding to phosphorylated β‐catenin, resulting in a dramatic reduction of β‐catenin ubiquitination. Of note, although proteasome inhibitor MG132 treatment inhibited degradation of phosphorylated β‐catenin, reduced association between Btrc exons 2 and 3, and phosphorylated β‐catenin was still observed (Figure 5D,E; Figure S7D,E, Supporting Information). In addition, we found that overexpression of short Btrc promoted the expression of *Snord15b* and Wnt target genes, suggesting a possible feedback regulation of the Wnt signaling pathway by the *Snord15b*‐Ilf2‐Btrc axis (Figure S7F, Supporting Information). In parallel, we established a culture system for intestinal organoids using ENR medium containing R‐spondin (R), epidermal growth factor (E), and Noggin (N). Then, we removed a Wnt pathway agonist R‐spondin from the medium and continued culturing organoids in EN medium for 12 h. We found that attenuation of Wnt signaling decreased expression of Wnt target genes and *Snord15b* (Figure S7G,H, Supporting Information). Ubiquitination of β‐catenin was remarkably elevated in intestinal organoids lacking *Snord15b* or *Ilf2* (Figure 5F; Figure S7I, Supporting Information). With cycloheximide (CHX) treatment, β‐catenin rapidly degraded in *Snord15 b‐*depleted organoids, whereas it remained more stable in control organoids. MG132 treatment prevented β‐catenin degradation (Figure S7J, Supporting Information). Similar results were observed in *Ilf2*‐deleted organoids (Figure S7K, Supporting Information). It is reported that Usp8 stabilizes the β‐catenin protein by inhibiting the K48‐specific poly‐ubiquitination process of the β‐catenin protein.^[^ ^27^ ^]^ We further investigated whether *Snord15b* also regulates the stability of β‐catenin by affecting other Wnt modulators. We found that the deletion of *Snord15b* neither affected the alternative splicing of Usp8 nor the protein level of Usp8 (Figure S7L,M, Supporting Information). These data suggest that the short isoforms of Btrc produced by *Snord15b*‐Ilf2 association inhibit ubiquitination of β‐catenin in ISCs.

β‐catenin serves as a key regulatory factor in Wnt signaling pathway. Upon activation of Wnt signaling, β‐catenin accumulates in the cytoplasm and subsequently translocates into the nucleus. Nuclear β‐catenin binds to TCF/LEF transcription factors and promotes transcription of downstream target genes.^[^ ^11^ ^]^ To examine the role of *Snord15b*‐Ilf2‐mediated Btrc splicing in Wnt signaling pathway, we generated MC38 cell lines lacking *Snord15b*, *Ilf2*, or the exon 2 and 3 of *Btrc* (△E2&3) (Figure S8A, Supporting Information). We observed that *Snord15b* KO or *Ilf2* KO caused decreased β‐catenin levels, while *Btrc* (△E2&3) increased β‐catenin levels, including non‐phosphorylated β‐catenin (Figure S8B, Supporting Information). Meanwhile, knockout of either *Snord15b* or *Ilf2* reduced expression and nuclear localization of β‐catenin. Conversely, *Btrc* (△E2&3) caused increased nuclear β‐catenin (Figure 5G; Figure S8C, Supporting Information), which was further confirmed by immunofluorescence staining with β‐catenin antibodies (Figure 5H). We then employed TOPflash/FOPflash assay to assess β‐catenin‐mediated transcriptional activity. We noticed that cells overexpressing the long isoform of Btrc exhibited decreased TCF/LEF transcriptional activity compared to those overexpressing the spliced short Btrc (Figure S8D, Supporting Information). Consistently, loss of *Snord15b* or *Ilf2* inhibited TCF/LEF transcriptional activity (Figure 5I). Additionally, we employed another cell line, CT26 cells, to assess the impact of *Snord15b*‐Ilf2‐Btrc on β‐catenin ubiquitination and nuclear localization. Results consistent with those of MC38 cells were obtained (Figure S8E–H, Supporting Information). Collectively, these findings indicate that the *Snord15b*‐Ilf2‐Btrc axis is involved in the regulation of activation of Wnt/β‐catenin signaling pathway in ISCs.

### 2.6 *Btrc* Knockout Enhances Stemness Maintenance of ISCs

**Figure 6:**
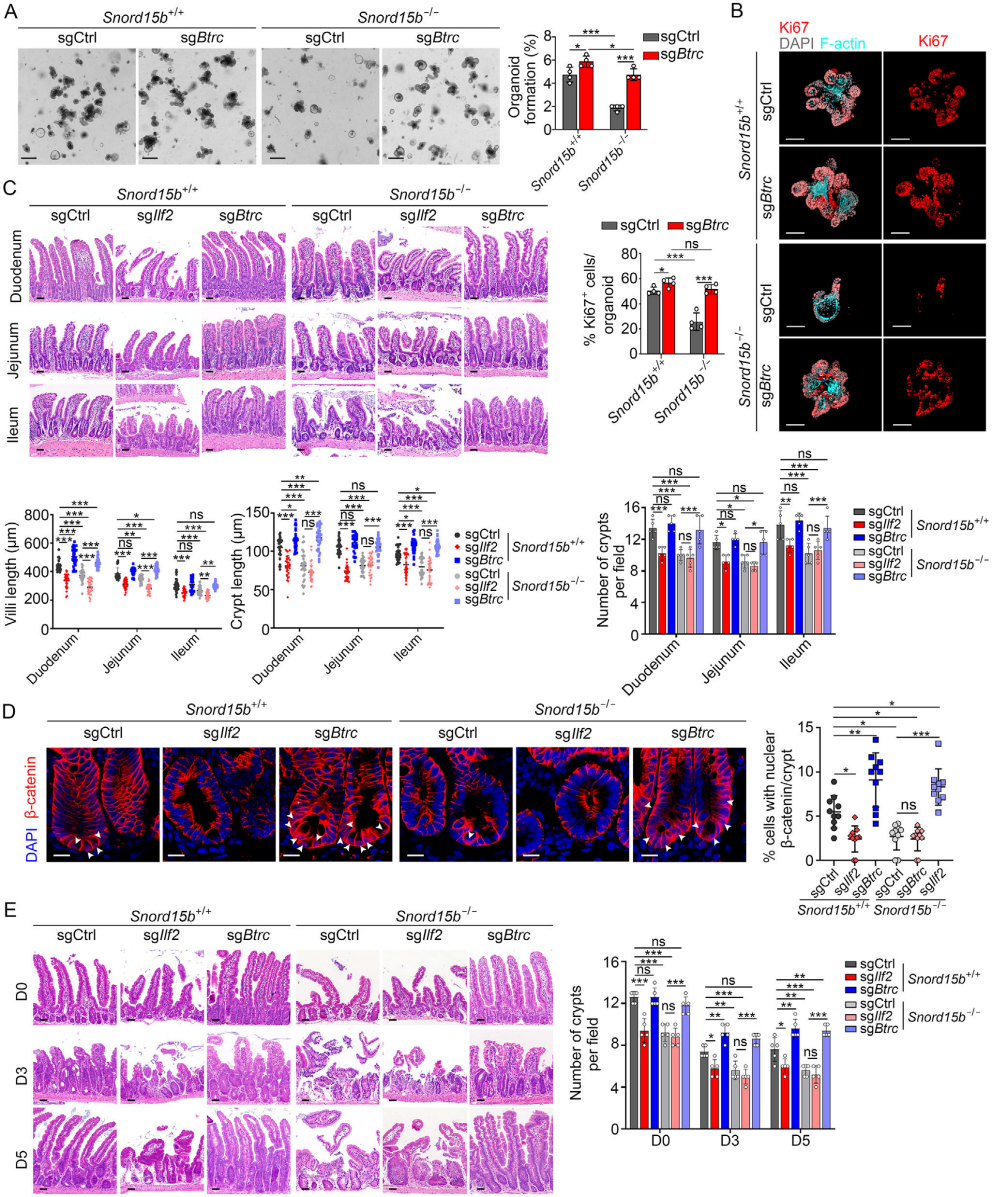
*Btrc* knockout enhances stemness maintenance of ISCs. A) Knockout of *Btrc* was conducted in *Snord15b* ^+/+^ and *Snord15b* ^−/−^ organoids using the CRISPR/Cas9 system, followed by an organoid formation assay. Scale bars, 300 µm. Organoid formation ratios per well are shown. *n* = 4 wells for each group. B) Immunofluorescence staining of Ki67 in *Btrc* KO and control organoids from *Snord15b* ^+/+^ and *Snord15b* ^−/−^ mice. Scale bars, 70 µm. Ratios of Ki67^+^ cells per organoid are shown in the left panel. *n* = 4 organoids for each group. C) Representative H&E staining images of three small intestine regions: duodenum, jejunum, and ileum from the indicated mice. Scale bars, 50 µm. Length of villi and crypts, as well as crypt numbers per field, are shown in the right panel. *n *= 30 villi or crypts for length calculation. *n* = 5 fields for crypt number calculation. D) Immunofluorescence visualization of β‐catenin expression in intestinal crypts from the indicated mice. Scale bars, 10 µm. In a crypt, proportions of cells with nuclear β‐catenin expression among all crypt cells were calculated and shown in the right panel. *n* = 10 crypts for each group. E) H&E staining of intestinal tissues from indicated mice at day 0, 3, 5 after 8 Gy of radiation damage. H&E staining results of the jejunum were shown as a representative. Scale bars, 70 µm. Crypt numbers per field are shown in the right panel. *n* = 5 fields were calculated for each group. Data are shown as the means ± SD. Statistical analysis was performed using one‐way (A,B,D) and two‐way ANOVA with Tukey's multiple comparisons test (C,E). *P < 0.05, **P < 0.01, ***P < 0.001, ns, not significant.

To further examine the physiological role of Btrc in the self‐renewal maintenance of ISCs, we employed CRISPR/Cas9‐mediated genome editing to delete *Btrc* in *Snord15b* ^+/+^ and *Snord15b* ^−/−^ ISCs (*Snord15b* ^+/+^;sg*Btrc* and *Snord15b* ^−/−^;sg*Btrc*). We found that *Btrc* deletion enhanced organoid formation of *Snord15b* ^+/+^ ISCs, whereas *Btrc* deletion in *Snord15b* ^−/−^ ISCs could restore diminished organoid formation capacity of *Snord15b* ^−/−^ ISCs (**Figure** **6**A). Subsequently, proliferative cells within organoids derived from *Btrc*‐deficient ISCs were dramatically increased (Figure 6B). We next generated *Btrc* or *Ilf2* deficient mice (*Snord15b* ^+/+^;sg*Ilf2*, *Snord15b* ^+/+^;sg*Btrc*, *Snord15b* ^−/−^;sg*Ilf2* and *Snord15b* ^−/−^;sg*Btrc*) via CRISPR/Cas9 system. As expected, Ilf2 deletion caused markedly shortened villi and crypts, accompanied by reduced crypts in small intestines of both *Snord15b* ^+/+^ and *Snord15b* ^−/−^ mice (Figure 6C). In contrast, *Btrc* deletion exhibited opposite effects. As showed in Figure 5H, *Btrc* deletion caused an increased nuclear β‐catenin in intestinal crypts of both *Snord15b* ^+/+^ and *Snord15b* ^−/−^ mice. Conversely, *Ilf2* deletion was associated with decreased nuclear translocation of β‐catenin (Figure 6D). Furthermore, we overexpressed β‐catenin in *Snord15b* ^−/−^ mice (Figure S9A, Supporting Information). We noticed that overexpression of β‐catenin promoted formation of intestinal organoids, increased lengths of small intestinal villi and crypts, elevated numbers of crypts and increased nuclear β‐catenin in intestinal crypts (Figure S9B–D, Supporting Information). We then assessed effects of *Btrc* or *Ilf2* deficiency on intestinal regeneration. Five days after irradiation induced epithelial injury (8 Gy), *Btrc* deletion enhanced intestinal regeneration in both *Snord15b* ^+/+^ and *Snord15b* ^−/−^ mice, while *Ilf2* deletion apparently impaired intestinal regeneration (Figure 6E). Taken together, Btrc is essential to the regulation of ISC stemness maintenance and intestinal regeneration.

## 3 Discussion

Proliferation and maintenance of ISCs are regulated by various niche factors, including Wnt, Notch, YAP/TAZ, and so on.^[^ ^28^ ^]^ In addition to microenvironmental signaling factors, our previous reports have identified several non‐coding RNAs that are involved in the regulation of self‐renewal of ISCs.^[^ ^29^, ^30^, ^31^ ^]^ In this study, we identified a novel stem cell‐associated small nucleolar RNA, *Snord15b*, which is highly expressed in ISCs. We found that *Snord15b* deficiency reduces the number of ISCs and impairs intestinal regeneration. Mechanistically, in WT ISCs, *Snord15b* associates with Ilf2 to recruit splicing factors for alternative splicing of *Btrc* mRNA, leading to the generation of a short isoform of *Btrc* mRNA. Short Btrc protein cannot form a functional E3 ubiquitin ligase complex for β‐catenin ubiquitination and subsequently stable β‐catenin translocates into the nucleus of ISCs for activation of the Wnt/β‐catenin signaling pathway, leading to ISC stemness maintenance and intestinal regeneration. In contrast, in *Snord15b*‐deficient ISCs, single Ilf2 cannot recruit splicing factors for the alternative splicing of *the* short isoform of *Btrc* mRNA, resulting in the generation of full‐length of *Btrc* mRNA and the formation of the contained E3 ligase complex for β‐catenin ubiquitination.

The Wnt/β‐catenin pathway comprises a major driving force for stem cell proliferation and maintenance, whose activity level is highest at the bottom of the intestinal crypt and decreases toward the villus region, forming a Wnt gradient. In our study, we found that *Snord15b* mediates ISC stemness via the Wnt signaling pathway. Conversely, the activity of the Wnt pathway affects *Snord15b* expression. Therefore, we proposed a feedback regulatory model: to maintain the homeostasis and stemness of ISCs, β‐catenin and phosphorylated β‐catenin are in a dynamic regulatory process. In ISCs located in the high‐concentration Wnt niche, increased β‐catenin promotes activation of the Wnt signaling pathway, which in turn enhances expression of *Snord15b* and promotes formation of short Btrc mediated by *Snord15b*‐Ilf2 to reduce ubiquitination and degradation of phosphorylated β‐catenin, allowing cells to maintain a low level of phosphorylated β‐catenin (Figure S9E, Supporting Information). In addition to regulating ISCs under a homeostatic condition, we found that *SNORD15B* is highly expressed in colon cancer cells, and it may be involved in intestinal tumorigenesis. Although phosphorylation of β‐catenin is blocked via multiple mechanisms in colon cancer, such as APC truncation and AXIN2 mutation, we found that CRC cell lines, such as MC38, CT26, and Caco‐2 cells exhibit phosphorylated β‐catenin. Due to multiple gene mutations and complex regulatory pathways of tumor cells,^[^ ^32^ ^]^ apart from the *Snord15b*‐Ilf2‐Btrc axis, the mechanism by which *Snord15b* stabilizes β‐catenin in cells lacking a functional β‐catenin destruction complex may involve other factors, which still need to be further exploration.

Usually, snoRNAs canonically modify rRNAs to exert their functions. However, a growing body of studies reveals that snoRNAs exert their effects in the regulation of stem cells independent of rRNA modifications. For instance, in leukemia stem cells, *SNORD118* and *SNORD3A* enrich and display a high frequency of trans‐association with chromatin. Suppression of *SNORD118* and *SNORD3A* impairs leukemia cell proliferation and colony‐forming capacity.^[^ ^33^ ^]^ *SNORD116* and *SNORD115* are highly accumulated during neuronal differentiation and are implicated in the pathogenesis of Prader‐Willi syndrome by influencing RNA stability and protein synthesis.^[^ ^34^ ^]^ Our previous work has demonstrated that *SNORD88B* regulates the self‐renewal of liver cancer stem cells by anchoring WRN in the nucleolus to activate Hippo signaling.^[^ ^35^ ^]^ However, the role of snoRNAs in ISC biology remains elusive. Here, we identified *Snord15b* as a key regulator of ISC stemness, which interacts with Ilf2 to mediate alternative splicing of *Btrc*, thereby enhancing β‐catenin stability to activate the Wnt/β‐catenin signaling pathway for ISC self‐renewal maintenance.

Besides classical binding proteins (FBL, NOP56, NOP58, SNU13), C/D‐box snoRNAs have been reported to interact with RNA‐binding proteins (RBPs) to exert their functions.^[^ ^35^ ^]^ Among these proteins, Ilf2, a multifunctional RBP, plays various roles in stem cells, including regulation of gene transcription, RNA splicing, stability, and transport. Ilf2 can act as a transcription factor. For example, lncRNA SNHG26 interacts with Ilf2 and relocates it from inflammatory genomic loci to the *LAMB3* locus to facilitate inflammatory‐to‐proliferative state transition of keratinocyte progenitors, enhancing tissue repair and regeneration.^[^ ^36^ ^]^ In addition, Ilf2 interacts with THO complex protein THOC4 to promote mRNA export of pluripotency transcription factors, resulting in enhanced stemness and tumor‐initiating capacity of esophageal cancer stem cells.^[^ ^37^ ^]^ Moreover, as a protein containing a DZF domain, Ilf2 has been reported to participate in the regulation of alternative splicing of mRNA, primarily controlling two types of splicing events: exon skipping and mutually exclusive splicing.^[^ ^38^ ^]^ In acute myeloid leukemia (AML), Ilf2 knockout decreases production of full‐length cereblon protein via modulating AS of CRBN mRNA, leading to diminished ubiquitination and proteasomal degradation of GSPT1, thereby decreases AML cell apoptosis and increases engraftment of leukemia stem cells.^[^ ^39^ ^]^ Although Ilf2 mRNA is widely expressed across various tissues, its role in intestinal homeostasis, particularly in the maintenance and differentiation of ISCs, remains unexplored. Here we found that *Snord15b*‐Ilf2 association mediates alternative splicing of *Btrc* to form a short Btrc protein, leading to disruption of the formation of Btrc contained E3 ligase for proteolysis of β‐catenin.

mRNA plays a critical regulatory role in proteome diversity, cellular fate, differentiation, maintenance of host homeostasis, and pathogenesis and progression of diseases.^[^ ^40^, ^41^ ^]^ Alternatively spliced mRNA isoforms are prevalent in all kinds of cells. Emerging evidence indicates that splicing factors are dynamically regulated in response to stem cell activities, suggesting that alternative splicing may serve as a critical regulatory mechanism in quiescent stem cells.^[^ ^42^ ^]^ However, little is known about the regulation and function of alternatively spliced isoforms in stem cells. Several reports have revealed the role of snoRNAs in the regulation of mRNA alternative splicing. *SNORD2* binds to an intronic sequence of EIF4A2 to conceal a branch point, leading to decreased inclusion of an adjacent alternative exon.^[^ ^43^ ^]^ Additionally, snoRNA HBII‐180C regulates alternative splicing of fibroblast growth factor receptor 3 (FGFR3) by masking splice sites, preventing inclusion of spliced exons.^[^ ^44^ ^]^ Here, we showed that disruption of *Snord15b*‐Ilf2 interaction remarkably affects the alternative splicing of the *Btrc* gene related to E3 ligase formation. We found that abnormal alternative splicing of *Btrc* caused by either *Snord15b* or Ilf2 deficiency promotes the production of full‐length Btrc protein to form a functional E3 ligase, resulting in excessive ubiquitination and degradation of β‐catenin, consequently inhibiting the Wnt signaling pathway in ISCs.

In summary, *Snord15b* is highly expressed in ISCs, which associates with Ilf2 to mediate alternative splicing of Btrc to form a short isoform of Btrc mRNA. The short Btrc protein fails to generate a Btrc‐containing E3 ligase for β‐catenin proteolysis, leading to activation of the Wnt/β‐catenin signaling pathway for ISC stemness maintenance and gut regeneration.

## 4 Experimental Section

### Antibodies and Reagents

Anti‐Ki67 (Cat# ab15580), Anti‐nucleolin (Cat# ab129200), Anti‐β‐catenin (Cat# ab32572), Anti‐Skp1 (Cat# ab76502), Anti‐non‐phospho‐β‐catenin (Cat# ab246504) and F‐actin staining kit (Cat# AB1112127) were all obtained from Abcam. Anti‐phospho‐β‐catenin (Ser33/37/Thr41) (Cat# 9561T), Anti‐Olfm4 (Cat# 39141S), and Anti‐Cleaved Caspase‐3 (Cat# 9661T) were purchased from Cell Signaling Technology. Anti‐OLFM4 (Cat# NBP2‐24535SS) was purchased from NOVUS. Anti‐EEA1 (Cat# sc‐137130) was from Santa Cruz. Anti‐β‐actin (Cat# A1978) and Anti‐Flag (Cat# F1804) antibodies were from Sigma‐Aldrich. Anti‐EpCAM (Cat# 118 212) and Anti‐GFP (Cat# 338 001) were from Biolegend. Anti‐Sfpq (Cat# A0958), Anti‐hnRNPA3 (Cat# A20212), and Anti‐K48‐linkage Specific Ubiquitin (Cat# A3606) were purchased from ABclonal. Anti‐Skp1 (Cat# K003423P), Anti‐Cul1 (Cat# K003246P), Anti‐BTRC (Cat# K002903P), Anti‐hnRNPA1 (Cat# K108413P), Anti‐PRPF6 (Cat# K111534P), Anti‐DDX5 (Cat# K112000P) and Anti‐Khdrbs1 (Cat# K000417P) were from Solarbio. Anti‐c‐myc (Cat# KM8003) was purchased from Sungene. Anti‐His (Cat# RM1001) was from Ray antibody. Anti‐LGR5 (Cat# MA5‐25644), Alexa‐594, Alexa‐488, and Alexa‐647 conjugated anti‐rabbit and anti‐mouse secondary antibodies were purchased from Invitrogen. Anti‐Ilf2 (Cat# 14714‐1‐AP), Anti‐hnRNPU (Cat# 16365‐1‐AP), and Anti‐HA (Cat# 51064‐2‐AP) were purchased from Proteintech. IntestiCult Organoid Growth Medium (Cat# 0 6005 (Mouse); 0 6010 (Human)) was purchased from STEMCELL Technologies. The Dual Luciferase Reporter Gene Assay Kit (Cat# RG027) was purchased from Beyotime. Biotin RNA Labeling Mix (Cat# 11 685 597 910), DIG RNA Labeling Mix (Cat# 11 277 073 910), and T7 RNA polymerase (Cat# 10 881 767 001) were from Roche. DeadEnd Fluorometric TUNEL System (Cat# G3250) was purchased from Promega. Paraformaldehyde (PFA) and 4′, 6‐diamidino‐2‐phenylindole (DAPI) were from Sigma‐Aldrich. Phosphatase Inhibitor Cocktail (Cat# PR20015) was from Proteintech. Protease Inhibitor Cocktail (Cat# 539 134) was purchased from Millipore. Cycloheximide (Cat# HY‐12320) was purchased from MCE. MG132 (Cat# 10 012 628) was purchased from Cayman. Recombinant murine EGF (Cat# 315‐09), recombinant murine R‐Spondin‐1 (Cat# 315–32), and recombinant murine Noggin (Cat# 250‐38) were purchased from PeproTech.

### Mice

*Snord15b* ^−^ *^/^* ^−^ mice were generated by CRISPR/Cas9 technology as described previously.^[^ ^35^ ^]^ A pair of single guide RNAs (sgRNAs) targeting the intron sequences flanking the *Snord15b* locus was designed. The corresponding sgRNAs are detailed in Table S1 (Supporting Information). In vitro transcribed Cas9 mRNA and sgRNAs were microinjected into zygotes from C57BL/6 mice. About 200–250 zygotes were implanted into the uterus of pseudo‐pregnant ICR females, from which viable founder mice were obtained. The following primers were used for deleted locus detection: 5′‐CAGGGTAAGTAGCTGAGTCCCTT‐3′ and 5′‐ACCTAGAAGACGCCAGGGTT‐3′.

For the generation of *Ilf2* and *Btrc* knockout mice, sgRNAs were designed on the CRISPR Guide RNA Design Tool (https://www.benchling.com/crispr), and corresponding sgRNA sequences are listed in Table S1 (Supporting Information). sgRNAs were cloned into LentiCRISPRv2 vector (Cat# 52 961, Addgene), then LentiCRISPRv2, pMD2G (Cat# 12 259, Addgene), and psPAX2 (Cat# 12 260, Addgene) plasmids were co‐transfected into HEK293T cells using Lipofectamine 3000. Cell supernatants were collected on the first, second, and third day after transfection and filtered through a 0.45 µm filter. Then supernatants containing virus particles were ultracentrifuged at 25 000 rpm for 2 h at 4 °C for viral concentration. The virus pellets were resuspended with PBS and injected i.v. into tamoxifen‐pretreated *Lgr5* ^GFP^; Cas9^KI^ mice. Virus injections were performed every other day and continued for two weeks.

Cas9‐KI and *Lgr5*‐EGFP‐IRES‐CreERT2 (*Lgr5* ^GFP^) mice were purchased from the Jackson Laboratory. All the mice were C57BL/6 background and 6–8 weeks old. Littermates were used with the same age and gender for each group. Animal use and protocols were approved by the Institutional Animal Care and Use Committees (approval number: SYXK2024221) at the Institute of Biophysics, Chinese Academy of Sciences.

### Human Intestinal Tissues and CRC Samples

Primary human CRC samples were collected from patients pathologically diagnosed with colorectal cancer from the Department of General Surgery, Peking University Third Hospital with informed consent and approval from the Ethics Committee of Peking University Third Hospital. The participants were selected randomly, without potential self‐selection bias or other biases.

### CRISPR/Cas9 Knockout System

*Snord15b, Ilf2*, and *Btrc* deletion cell lines and intestinal organoids were established using CRISPR/Cas9 technology provided by Zhang's laboratory. sgRNAs were designed and cloned into the LentiCRISPRv2 vector (Cat# 52 961, Addgene), then lentivirus packaging, collection, and concentration were performed as above. For gene editing in intestinal organoid cells, organoids were broken into small fragments and resuspended with 500 µL organoid medium mixed with lentivirus solution and 6 mg mL^−1^ polybrene (Sigma‐Aldrich). The mixture was transferred into 24‐well plates for centrifugation at 32 °C, 600 × g, for 1 h, following cultured at 37 °C for 6 h. After that, cells were recollected and centrifuged to remove virus and resuspended with Matrigel for organoid culture. Change the culture medium every two days. After several days, 1 mg mL^−1^ puromycin was added into the medium for selection. For gene editing in cell lines, after lentivirus infection, a puromycin‐selected single cell was seeded in one well of a 96‐well plate and cultured for expansion. The knockout of the target gene was validated by qRT‐PCR or Western blotting.

### RNA Interference and Gene Overexpression Systems

RNA interference was performed based on short hairpin RNA.^[^ ^45^ ^]^ Target sequences are listed in Table S2 (Supporting Information). shRNAs were cloned into the pSicor‐Puro lentivirus vector. The lentiviral vectors were co‐transfected with psPAX2 and pMD2G into HEK293T cells for viral packaging. Lentiviral pellets were obtained, and the following organoid infection was performed as described above. The infected organoids were passaged and 1 mg mL^−1^ puromycin was added into the medium for selection. RNA interference efficiency was evaluated by qRT‐PCR.

For gene overexpression, genes were constructed into the pLVX‐IRES‐Puro lentivirus vector. Lentiviral vectors were co‐transfected with psPAX2 and pMD2G into HEK293T cells. Virus packaging, collection, and organoids infection, selection, as well as efficiency analysis were performed as above.

### Isolation of Intestinal Crypts and Organoids Formation

Intestinal crypts were isolated and cultured as previously described with minor modifications.^[^ ^46^ ^]^ For isolation of intestinal crypts, 8‐week mice were sacrificed. Small intestines were opened longitudinally and washed with pre‐cooled PBS. Then intestines were cut into 0.1 cm pieces and incubated in collagenase solution (DMEM/F12 medium containing 0.1% type I collagenase (Invitrogen), 100 units mL^−1^ penicillin, 0.1 mg mL^−1^ streptomycin, and 10 mm HEPES) at 37 °C for 30 min. The mixture obtained after washing and repeatedly blowing was then passed through a 70 µm cell strainer (BD Biosciences) and centrifuged at 70 × g for 5 min to collect intestinal crypts. To isolate Lgr5⁺ ISCs and Lgr5^−^ IECs, crypts were separated from *Lgr5* ^GFP^ mice as previously described. The crypts were dissociated into single cells using TrypLE Express supplemented with 0.8 kU mL^−1^ DNase I (Roche). GFP⁺ and GFP^−^ cells were sorted by flow cytometry and used as Lgr5⁺ ISCs and Lgr5^−^ IECs in subsequent experiments. For isolation of human LGR5⁺ ISCs, crypts from human colonic tissues were separated and dissociated into single cells following the same procedure. Cells were then stained with an anti‐LGR5 antibody on ice for 30–40 min, washed with PBS containing 1% FBS, and LGR5⁺ ISCs were sorted via flow cytometry.

For intestinal organoids culture, crypts were embedded in Matrigel and seeded on a six‐well plate. After polymerization, IntestiCult Organoid Growth Medium (STEMCELL Technologies) was added and refreshed every two days. For passaging, the medium was removed, and Cell Recovery Solution (Corning) was added to release organoids from Matrigel. Then organoids were broken into small fragments, centrifugated at 70 × g for 5 min, and resuspended in fresh Matrigel and seeded on the new six‐well plate with fresh medium added. For organoids culture with ENR medium, the culture medium (Advanced DMEM/F12 containing growth factors (10–50 ng mL^−1^ EGF), 500 ng mL^−1^ R‐spondin‐1, and 100 ng mL^−1^ Noggin) was prepared and added.

### Quantitative Real‐Time PCR

Samples were homogenized directly into TRIzol (QIAGEN), and RNA was extracted via chloroform extraction. The quantity and quality of RNA were determined by using a NanoDrop (Thermo Fisher). cDNA was synthesized with 5 × All‐In‐one RT Mastermix (Abm, Vancouver, Canada) and analyzed on the QuantStudio1 qPCR system using specific primer pairs listed in Table S3 (Supporting Information). Relative expression was calculated and normalized to endogenous U6 or *Actb*.

### Northern Blotting

Total RNA was extracted with TRIzol methods, then subjected to electrophoresis on a urea‐polyacrylamide gel for 2.5 h. Then, RNA was transferred to a positively charged nylon membrane with 0.5 × TBE buffer. Membranes were cross‐linked under 265 nm UV with an energy of 240 000 µJ cm^−2^ and pre‐hybridized, following incubated with biotin‐labeled RNA probes at 65 °C for 16–20 h. After washing, biotin signals were detected with Chemiluminescent Nucleic Acid Detection Module (Cat# 89 880, Thermo) according to the manufacturer's instructions.

### Biotin‐Labeled RNA Pulldown and Mass Spectrometry Assay

Biotin‐labeled RNAs were in vitro transcribed using Biotin RNA Labeling Mix and T7 RNA polymerase. Intestinal crypts were isolated and lysed with RIPA lysis buffer (strong) (GenStar) at 4 °C for 1 h. The lysates were incubated with biotin‐labeled probes overnight at 4 °C, then streptavidin‐conjugated agarose beads were added for enrichment of RNA‐binding proteins. After 4 h of incubation, biotin‐enriched proteins were separated by SDS–PAGE, and visualized by silver staining. Differential bands enriched by *Snord15b* were analyzed by mass spectrometry (Q‐Exactive, Thermo).

### Fluorescence In Situ Hybridization

For *Snord15b* in situ hybridization, intestinal sections or ISCs were fixed with 4% PFA and permeabilized with 1% TritonX‐100 for 30 min, then incubated with biotin‐labeled probes diluted in FISH hybridization buffer (50% formamide, 2 × SSC, 0.5 mg mL^−1^ yeast transfer RNA, 0.5 mg mL^−1^ salmon sperm DNA, 2.5 mg mL^−1^ bovine serum albumin) at 55 °C for 1 h. After washing with SSC washing buffer, the Fluorescent in Situ Hybridization Kit (RiboBio) was used for subsequent processing. Stained sections were mounted with Fluormount G (Southern Biotech) and visualized with confocal microscopy (Nikon, A1R+).

### Immunofluorescence Staining

For staining of sections, small intestines or colons were collected, opened longitudinally, and washed with PBS, followed by fixation with 4% PFA for 1 h. The samples were then dehydrated by a 30% sucrose solution for 24 h and embedded in OCT freezing media for sectioning. Tissue sections were briefly rehydrated in PBS and then permeabilized with 1% Triton X‐100 solution (PBS diluted) for 1 h, followed by blocking in 10% donkey serum (diluted in PBS) for 30 min. Blocked sections were incubated with appropriated primary antibodies overnight at 4 °C, and then incubated with fluorescence‐conjugated secondary antibodies at room temperature for 1 h. Mounted sections were visualized with confocal microscopy. Images were analyzed by Imaris 9 software. Immunofluorescence staining was performed to three intestinal segments (duodenum, jejunum, and ileum) and presented immunofluorescence staining images of the jejunum as a representative in the figures.

### Western Blotting

ISCs, crypts, or organoids were lysed with RIPA lysis buffer (strong) at 4 °C for 30 min and centrifugated at 12 000 × g for 10 min to collect supernatants. The sample supernatants were then separated with SDS‐PAGE, and the proteins were transferred to nitrocellulose membranes (Bio‐Rad). The membranes were incubated with primary antibodies overnight, followed with washing three times using Tris‐buffered saline with Tween‐20 (TBST), then the membranes were incubated with HRP‐conjugated secondary antibodies and visualized by chemiluminescent substrate (Thermo).

### Isolation of Nucleoplasmic, Cytoplasmic, and Nucleoli Fractions

Nucleoplasmic, cytoplasmic, and nucleoli fraction isolation were performed as previously described with minor modifications.^[^ ^47^ ^]^ Briefly, crypts were isolated from mouse intestines and dissociated into single cells. Cells were washed by pre‐cooled PBS and incubated in NSB (10 mm Tris‐Cl, pH 7.4, 10 mm NaCl, 0.5–2 mm MgCl_2_) at 4 °C for 30 min. Then appropriate volume of 10% NP‐40 solution was added in the homogenates to obtain a final concentration of 0.3%. The homogenates were transferred into 15‐mL Dounce homogenizers for the separation of nuclei and cytoplasm. After fifteen strokes with the Dounce pestles, the mixtures were taken to microscopy and the nucleus appeared no plasma membrane remnants. Homogenates were centrifugated at 1200 × g, 4 °C for 5 min. The supernatants were collected as the cytoplasmic fraction. The pellets were resuspended with 250 mm sucrose solution containing 10 mm MgCl_2_, then 880 mm sucrose containing 5 mm MgCl_2_ was added below. After centrifuging at 1200 × g, 4 °C for 10 min, pellets were resuspended with 340 mm sucrose solution containing 5 mm MgCl_2_ and broken by sonicating using 8 bursts of 10 s at 400 V with 10 s intervals. Sucrose (880 mm) was added under the sonicated nuclei, followed by centrifugation at 2000 × g, 4 °C for 20 min. The supernatants were nucleoplasmic fractions, and the pellets were nucleoli.

### Electrophoretic Mobility Shift Assay (EMSA)

Biotin‐labeled or unlabeled *Snord15b* were in vitro transcribed using Biotin RNA Labeling Mix or DIG RNA Labeling Mix, and T7 RNA polymerase. Recombined Ilf2 protein and *Snord15b* were incubated in EMSA binding buffer and conducted mobility shift assay using native gel electrophoresis. Biotin signals were detected according to the Chemiluminescent Nucleic Acid Detection Module.

### Immunohistochemistry Assay

Paraffin sections of intestinal tissues were deparaffinized in xylene and rehydrated in gradient alcohol, and treated with 3% hydrogen peroxide (H_2_O_2_) for 10 min to block endogenous peroxidase activity. Antigen retrieval was performed by heating the sections in boiling Tris‐EDTA buffer for 15 min. After blocked with 10% donkey serum for 30 min at room temperature, the sections were incubated with primary antibodies at room temperature for 2 h. Followed by incubating with HRP‐conjugated secondary antibodies for an additional hour, the sections were performed with DAB, counterstained with hematoxylin, and then dehydrated and mounted for microscopic visualization.

### Analysis of 2′‐O‐Methylation on 28S‐A3441

2′‐O‐methylation of 28S‐A3441 was analyzed by RTL‐P assay with minor modifications.^[^ ^20^, ^48^ ^]^ In brief, the primer used in the reverse transcription (RT) reaction was designed downstream of the predicted 2′‐O‐methylated site. Two forward primers for the subsequent PCR amplification were designed and located either downstream (*F*_D_) or upstream (*F*_U_) of the predicted 2′‐O‐methylated site. Reverse transcription was performed using M‐MLV reverse transcriptase (Promega), RNase inhibitor (Mei5bio), and 1 mm of RT primer, with either 10 or 1 mm dNTPs in the reaction. The reaction was performed at 37 °C for 5 min and at 70 °C for 15 min. Amplification of cDNA with *F*_U_/*R* and *F*_D_/*R* was quantified by qRT‐PCR, and methylation levels were analyzed by *F*_U_ *R*/*F*_D_ *R*. The sequence of primers *F*_U_, *F*_D_, and *R* is listed in Table S3 (Supporting Information).

### Domain Mapping

According to the Ilf2 functional domain regions, Ilf2 was divided into three fragments, which were constructed into 3 × Flag plasmids. Plasmids were transfected into HEK293T cells using Lipofectamine 3000. After 2 days, cell lysates were collected and performed RNA pulldown and Western blotting.

### TOPFlash Luciferase Assay

Wnt/β‐catenin‐specific TOPFlash reporter (Cat# D2501) and mutant FOPFlash reporter plasmids (Cat# D2503) were purchased from BeyoTime. TOPFlash and FOPFlash along with the pRL‐TK reporter plasmid were transfected into the indicated cells using Lipofectamine 3000. After 36 h, cells were lysed and detected according to the Dual Luciferase Reporter Gene Assay Kit (Cat#RG027, BeyoTime). Wnt/β ‐catenin activation levels were measured by TOPFlash/FOPFlash ratio.

### Immunoprecipitation Assay

For endogenic immunoprecipitation, organoids or crypts were lysed with RIPA buffer on ice for 1 h. Lysates were incubated with the indicated antibodies for 2 h and immunoprecipitated with protein A/G agarose beads for 1 h, followed by SDS‐PAGE separation and Western blotting.

### Minigene Assays

Minigene assays were based on the pSPL3 exon trapping vector. Cloned inserts included the second and third exons of *Btrc* with a variable length of flanking 5′ and 3′ intronic sequences. The minigene constructs were transfected into *Snord15b* KO, *Ilf2* KO, and control MC38 cells. After 36 h, RNA was extracted with the TRIzol regent followed by reverse transcription. The resulting cDNA was amplified with primers SD6: 5′‐TCTGAGTCACCTGGACAACC‐3′ and SA2: 5′‐ATCTCAGTGGTATTTGTGAGC‐3′.

### SnoRNA and Transcriptome Sequencing

For snoRNA sequencing, crypts were isolated from the small intestines of 8‐week *Lgr5* ^GFP^ mice, then Lgr5^+^ ISCs and Lgr5^−^ IECs were sorted by flow cytometry. Total RNA was extracted with TRIzol reagent and snoRNAs were sequenced using a mouse snoRNA Array (YINGBIOTECH). For identification of *Snord15b* downstream target genes, *Snord15b^+/+^* and *Snord15* ^−^ *^/^* ^−^ Lgr5‐GFP^+^ ISCs were sorted, then total RNA was extracted and subjected to sequencing analysis (Beijing Cnkingbio).

### Alternative Splicing Analysis

Aligned mapped reads of RNA‐Seq data were used for AS analysis. AS events were identified by rMATS (version 4.1.0), in which percent‐spliced‐in (PSI) for each splicing event was calculated based on the number of sequencing reads supporting the inclusion and exclusion isoforms, respectively. AS events were classified into skipped exons, retained introns, alternative 5′ splice sites, alternative 3′ splice sites, and mutually exclusive exons. Significant events were filtered out with FDR < 0.05 and | deltaPSI | > 0.1.^[^ ^49^ ^]^ Sequences for the primers used to confirm alternative splicing are provided in Table S4 (Supporting Information).

### Statistics and Reproducibility

Sample sizes, replicates along with statistical tests were reported in each figure legend. For statistical evaluation, data were analyzed by GraphPad Prism 8.0. Two‐tailed unpaired Student's *t*‐test and other appropriate statistical methods were used in this study. Results were shown as means ± SD. *P*‐values ≤ 0.05 were considered significant (*P < 0.05; **P < 0.01; ***P < 0.001) and *P* > 0.05 was considered non‐significant (ns). All flow cytometry data were analyzed by FlowJo 10.

## Conflict of Interest

The authors declare no conflict of interest.

## Author Contributions

Y.X., P.Z., Z.X., and Y.L. contributed equally to this work. Y.X. designed the project, performed experiments, analyzed data, and wrote the paper. P.Z., Z.X., Y.L., and H.F. performed experiments. D.F. and X.Z. constructed genetic mouse strains. Y.X., Z.X., P.Z., H.G., Z.W., R.W., C.L., S.Z., and Y.D. analyzed data. Y.T. generated animal models and analyzed data; Z.F. initiated the study, organized, designed, and wrote the paper

## Supporting information



Supporting Information

## Data Availability

Research data are not shared.
