# Supplementary material for: Snord15b Maintains Stemness of Intestinal Stem Cells via Enhancement of Alternative Splicing of Btrc Short Isoform for Suppression of β‐Catenin Ubiquitination
Source: Adv Sci (Weinh). 2025 Aug 30;12(44):e04485. doi: 10.1002/advs.202504485 (PMC12667501; doi:10.1002/advs.202504485)
Supplement: Supplementary file 1 — Supporting Information [file ADVS-12-e04485-s001.docx]

**Appendix for:**

***Snord15b* maintains stemness of intestinal stem cells via enhancement of alternative splicing of *Btrc* short isoform for suppression of β-catenin ubiquitination**

Yuwei Xu, Peikang Zhang, Zhen Xiong, Yufei Lan, Hui Guo, Runyuan Wu, Cunzhen Li, Hongzhe Fan, Ying Du, Xiaoxiao Zhu, Dongdong Fan, Zhonglong Wang, Yong Tian, Zusen Fan

**Supplementary Figures**

Figure S1. Biological characteristics of *Snord15b*.

Figure S2. Generation of *Snord15b*^−/−^ mice and *Snord15b* KO impairs ISC self-renewal maintenance.

Figure S3. Detection of canonical function of *Snord15b*.

Figure S4. Interaction of ILF2 with *SNORD15B* enhances stemness of human intestinal ISCs.

Figure S5. Alternative splicing of genes associated with ubiquitin mediated proteolysis.

Figure S6. Deletion of *Snord15b* or Ilf2 increases full length of Btrc.

Figure S7. Deletion of *Snord15b* or Ilf2 promotes the ubiquitination and degradation of β-catenin.

Figure S8. Deletion of *Snord15b* or Ilf2 inhibits Wnt/β-catenin signaling activation.

Figure S9. β-catenin overexpression enhances stemness maintenance of ISCs.

**Supplementary Tables**

Table S1. sgRNA sequences used in this study.

Table S2. shRNA sequences used in this study.

Table S3. Primer sequences used for qPCR.

Table S4. Primer sequences used for semi-quantitative PCR.


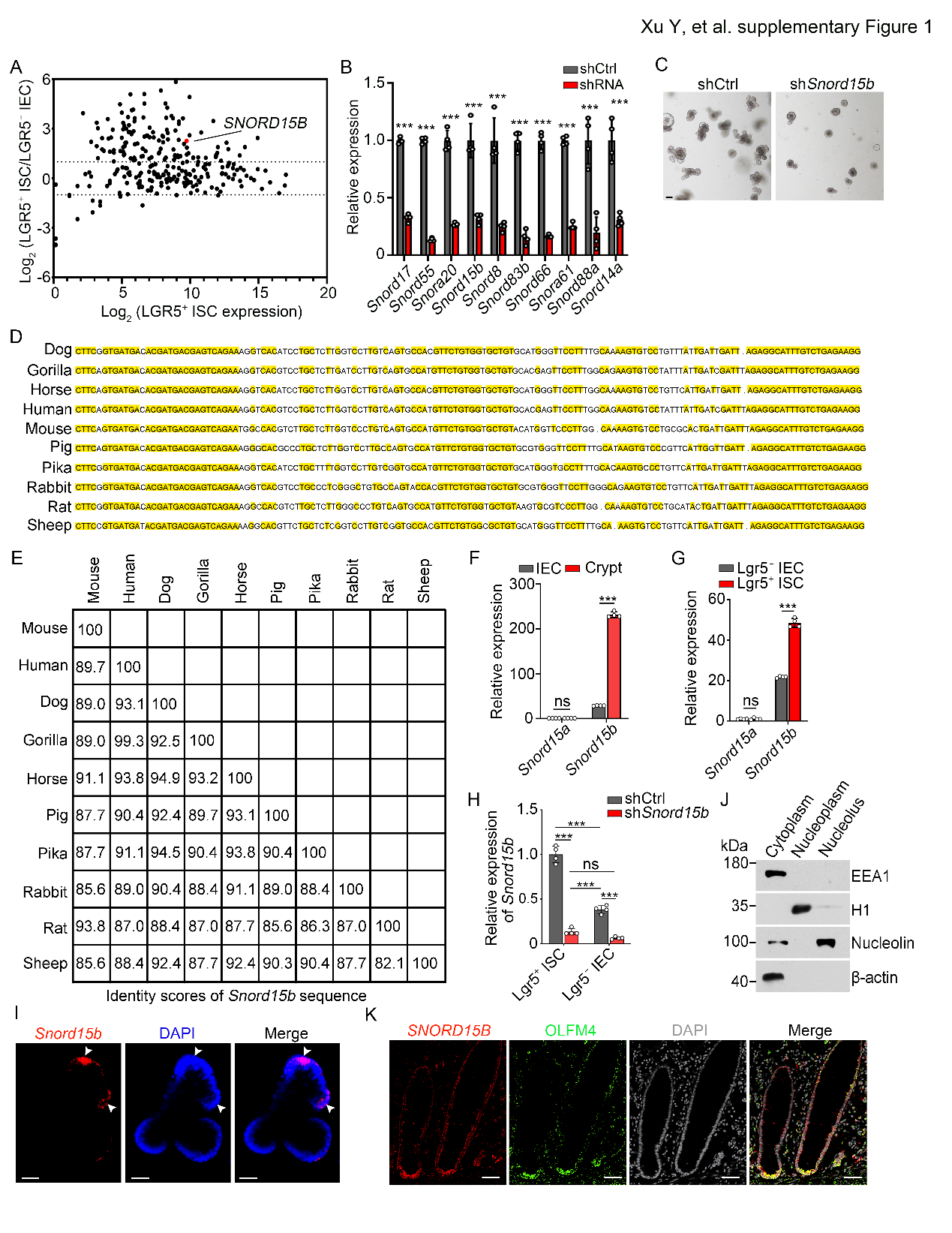


**Figure S1. Biological characteristics of *Snord15b*.** (A) LGR5^+^ and LGR5^−^ cells were sorted from human intestine tissues and performed snoRNA sequencing. Data are shown as a scatter plot of snoRNA in LGR5^+^ and LGR5^−^ cells and *SNORD15B* is marked in red. (B) Knockdown efficiency of top 10 upregulated snoRNAs with high abundance in ISCs was examined by qRT-PCR. n = 4 biologically independent samples. (C) Organoids images of shCtrl or sh*Snord15b* are shown. Scale bars, 100 μm. (D) Sequence alignment of *Snord15b* among different species. Conserved sequences are marked in yellow. (E) Comparison of sequence similarities of *Snord15b* among different species. (F) Relative mRNA expression levels of *Snord15b* and *Snord15a* in IEC and crypts. n = 4 independent experiments. Relative expression of *Snord15a* in IEC was used as a reference. (G) Relative mRNA expression levels of *Snord15b* and *Snord15a* in Lgr5^+^ and Lgr5^-^ cells. n = 4 independent experiments. Relative expression of *Snord15a* in Lgr5^-^ cells was used as a reference. (H) Relative mRNA expression levels of *Snord15b* in Lgr5^+^ and Lgr5^-^ cells with or without *Snord15b* silencing. n = 4 independent experiments. Relative expression of *Snord15b* in control Lgr5^+^ cells was used as a control. (I) *Snord15b* was visualized by FISH in small intestinal organoids. Scale bars, 50 μm. (J) Western blotting analysis of proteins from cellular subfractions. EEA1, H1 and nucleolin are protein markers for cytoplasm, nucleoplasm and nucleolus, respectively. (K) *SNORD15B* was visualized by FISH in human intestine tissues. Scale bars, 50 μm. OLFM4 (green) was used to mark ISCs. Data are shown as the means ± SD. Statistical analysis was performed using one-way with Tukey’s multiple comparisons test (H); comparisons two groups for a single variable were performed via multiple unpaired Student's t-tests with Holm-Šidák correction (B, F and G). ***P < 0.001, ns, not significant.


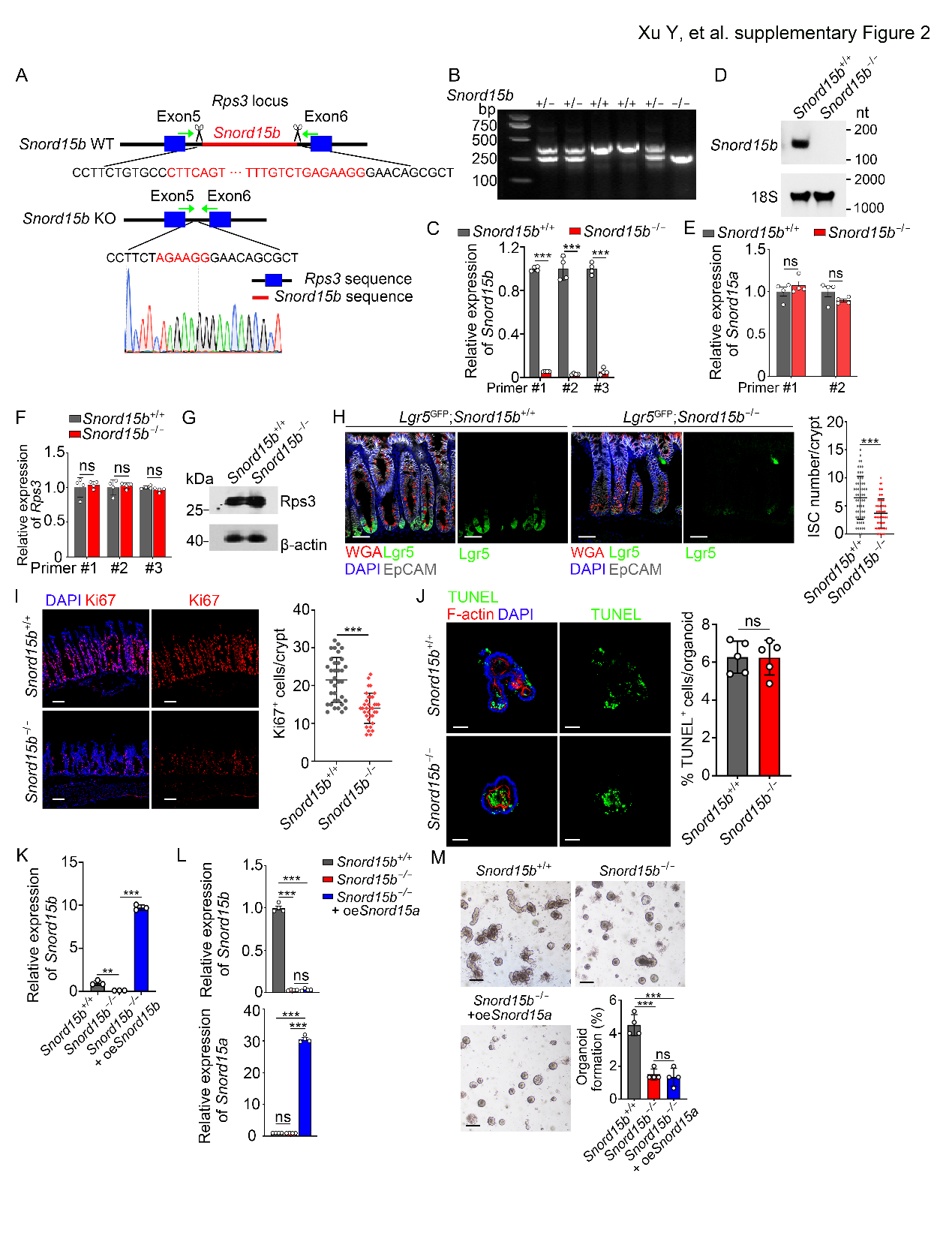


**Figure S2. Generation of** ***Snord15b*^−/−^ mice and *Snord15b* KO impairs ISC self-renewal maintenance.** (A) Scheme for *Snord15b* knockout mice. Gene locus of S*nord15b* was deleted using CRISPR/Cas9 system. (B) *Snord15b*^−/−^ mice were identified by agarose gel electrophoresis. (C-D) qRT-PCR (C) and Northern blotting (D) were used to determine *Snord15b* mRNA levels in *Snord15b*^+/+^ and *Snord15b*^−/−^ ISCs. n = 4 mice for each group. (E-F) Relative mRNA levels of *Snord15a* (E) and *Rps3* (F) in *Snord15b*^+/+^ and *Snord15b*^−/−^ ISCs were examined by qRT-PCR. Data were normalized to endogenous *Actb*. n = 4 mice for each group. (G) Rps3 protein levels in *Snord15b*^+/+^ and *Snord15b*^−/−^ ISCs were detected by Western blotting. (H) Lgr5^+^ ISCs from colons of *Lgr5*^GFP^; *Snord15b*^+/+^ and *Lgr5*^GFP^; *Snord15b*^−/−^ mice were visualized and calculated. Scale bars, 50 μm. Statistical ISC numbers per crypt are shown in right panel. n = 60 crypts for each group. (I) Immunofluorescence staining of Ki67 in *Snord15b*^+/+^ and *Snord15b*^−/−^ mouse colon sections. Scale bars, 100 μm. Numbers of Ki67^+^ cells per crypt are shown in right panels. n = 35 crypts for each group. (J) Immunofluorescence staining of apoptotic cells expressing TUNEL in *Snord15b*^+/+^ and *Snord15b*^−/−^ intestinal organoids. Scale bars, 50 μm. Ratios of TUNEL^+^ cells per organoid are shown in right panel. n = 5 organoids for each group. (K) Overexpression of *Snord15b* in *Snord15b*^−/−^ ISCs was validated by qRT-PCR. n = 3 independent experiments. (L) Overexpression of *Snord15a* in *Snord15b*^−/−^ ISCs was validated by qRT-PCR. n = 4 independent experiments. (M) Organoid formation was conducted in *Snord15b*^+/+^, *Snord15b*^−/−^ and *Snord15b*^−/−^ + oe*Snord15a* ISCs. Lentivirus was used for overexpression of *Snord15a*. Scale bars, 200 μm. Ratios of organoid formation per well were calculated and shown in lower panel. n = 4 wells for each group. Data are shown as the means ± SD. Statistical analysis was performed using unpaired two-tailed Student’s t-tests (I, J and L) and one-way ANOVA with Tukey's multiple comparisons testing (K and M); comparisons two groups for a single variable were performed via multiple unpaired Student's t-tests with Holm-Šidák correction (C-F). *P < 0.05, **P < 0.01, ***P < 0.001, ns, not significant. **P < 0.01, ***P < 0.001, ns, not significant.


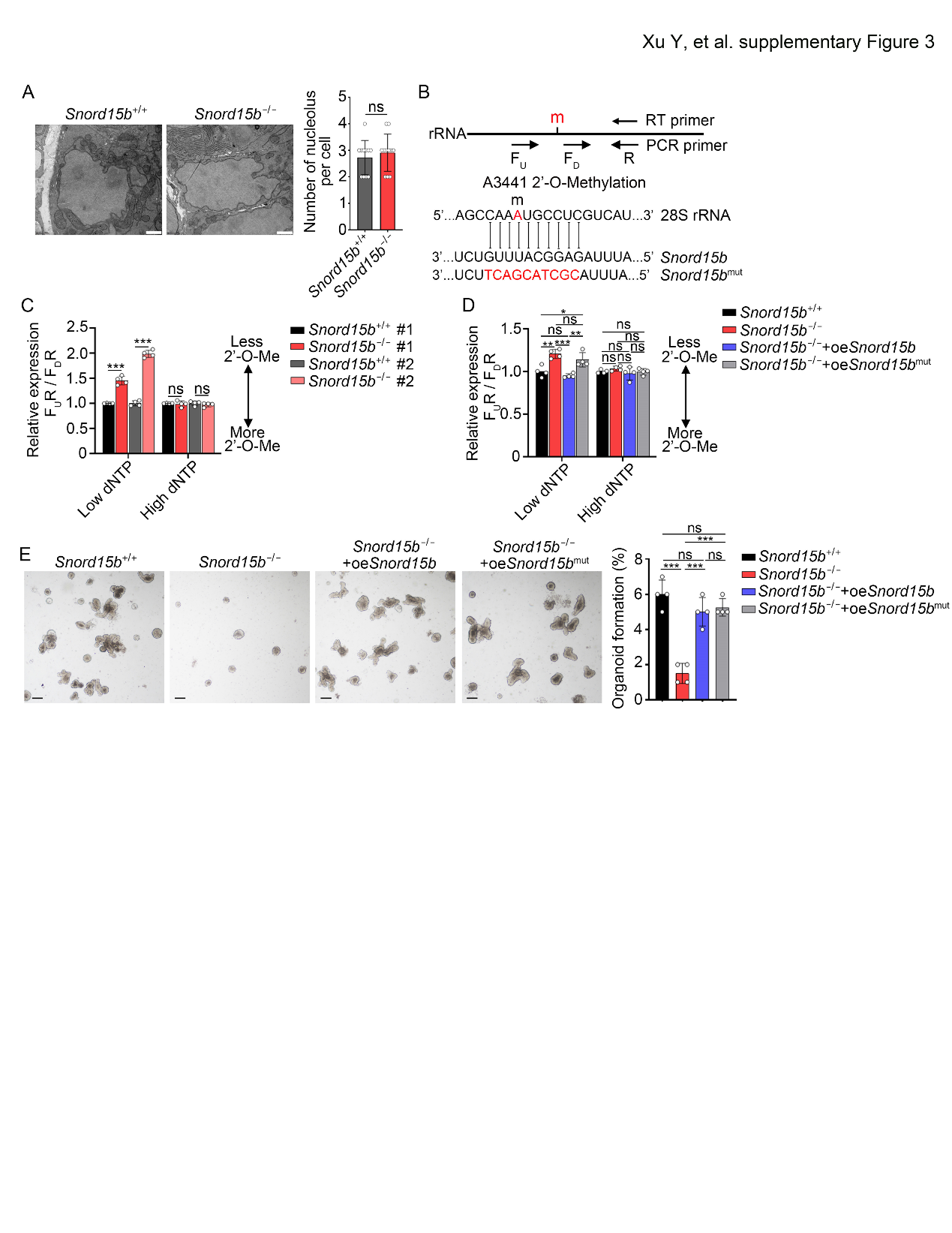


**Figure S3. Detection of canonical function of *Snord15b*.** (A) Electron microscope images of *Snord15b*^+/+^ and *Snord15b*^−/−^ Lgr5^+^ ISCs. Scale bars, 1 μm. Numbers of nucleoli per ISC are shown in right panel. n = 11 ISCs for each group. (B) Diagram for prediction of *Snord15b* binding regions with 28S rRNA via snOPY website. A3441 is predicted Nm target (red). Illustration of RTL-P method to analyze *Snord15b*-mediated 2’-O-methylation of 28S rRNA. (C) Amounts of F_U_R and F_D_R products from low or high dNTP reactions in *Snord15b*^+/+^ and *Snord15b*^−/−^ ISCs were detected by qRT-PCR. Relative expression of F_u_R/F_D_R in *Snord15b*^−/−^ ISCs was compared to their control samples. Higher F_u_R/F_D_R expression means less *Snord15b*-mediated 2’-O-methylation of 28S rRNA. n = 4 independent experiments. (D) Lentivirus was used for overexpressing *Snord15b* or *Snord15b*^mut^ (lack of binding region of *Snord15b*) in *Snord15b*^−/−^ ISCs, followed by organoid formation. Levels of 2’-O-methylation at 28S-A3441 in *Snord15b*^−/−^, *Snord15b*^−/−^ + oe*Snord15b*^WT^ and *Snord15b*^−/−^ + oe*Snord15b*^mut^ organoids were analyzed and compared to *Snord15b*^+/+^ organoids. n = 4 independent experiments. (E) Images of *Snord15b*^+/+^, *Snord15b*^−/−^, *Snord15b*^−/−^ + oe*Snord15b*^WT^ and *Snord15b*^−/−^ + oe*Snord15b*^mut^ organoids. Scale bars, 100 μm. Ratios of organoid formation per well were calculated and shown in right panel. n = 4 wells for each group. Data are shown as means ± SD. Statistical analysis was performed using unpaired two-tailed Student’s t-tests (A), one-way (E) and two-way ANOVA with Tukey’s multiple comparisons test (C and D). *P < 0.05, **P < 0.01, ***P < 0.001, ns, not significant.


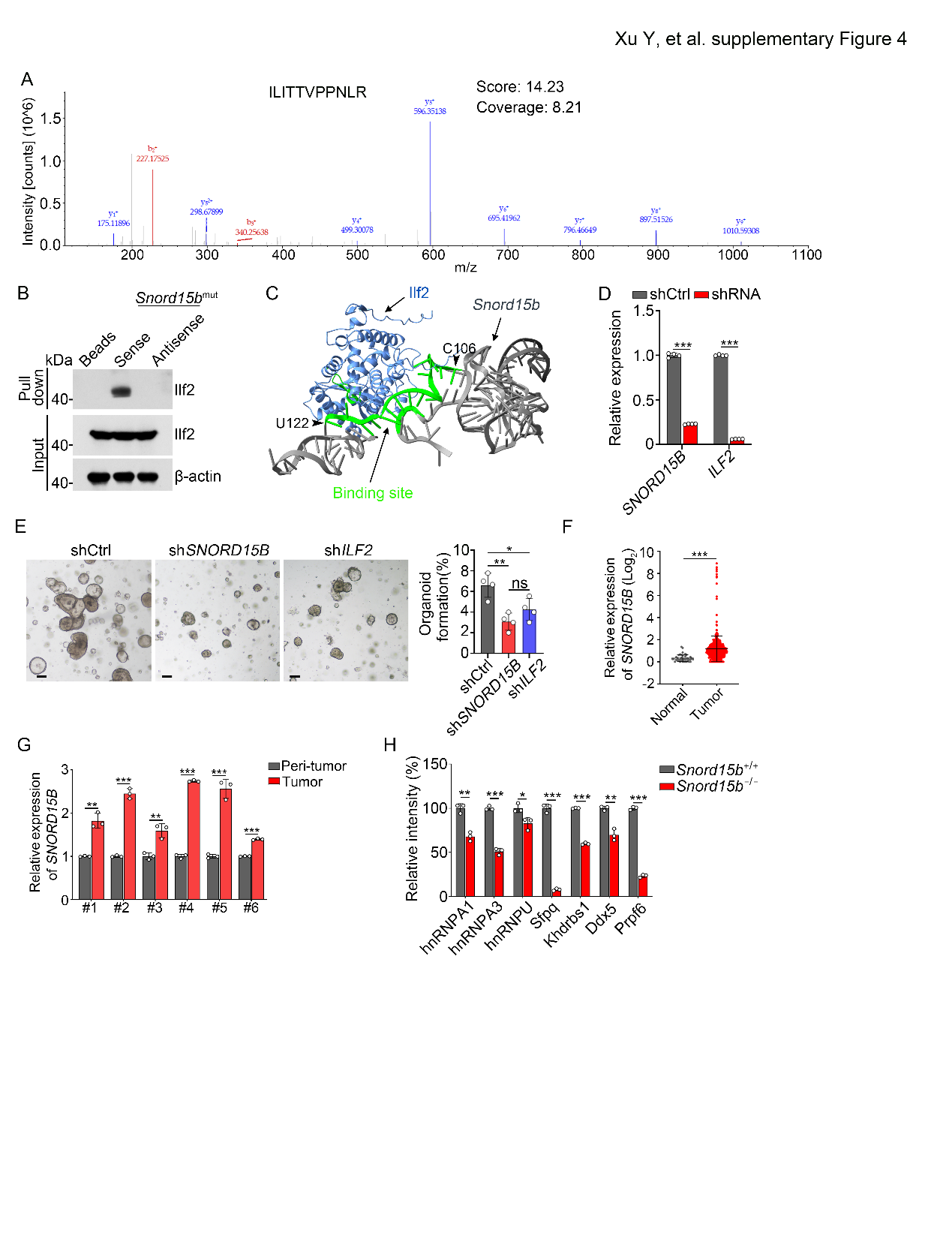


**Figure S4. Interaction of ILF2 with *SNORD15B* enhances stemness of human intestinal ISCs.** (A) MS profiles of Ilf2, corresponding peptide sequences were listed on the top of diagram. (B) Intestinal crypts from WT mice were lysed and incubated with mutated biotin-labeled *Snord15b*, followed by RNA-pulldown assay. Interaction of Ilf2 with mutated *Snord15b* was validated by Western blotting. (C) *Snord15b* was predicted to interact with Ilf2 via HDOCK website. Binding sites of *Snord15b* were marked by green color. (D) *SNORD15B* or *ILF2* was silenced in cells from human colonic crypt via shRNA, followed by organoid formation assay. Silencing of *SNORD15B* or *ILF2* in indicated organoids was validated by qRT-PCR. *SNORD15B* and *ILF2* expression was normalized to endogenous *U6* and *ACTB* respectively. n = 4 biologically independent samples. (E) Organoid formation of human colonic crypt with *SNORD15B* or *ILF2* silencing. Scale bars, 200 μm. Ratios of organoids formation per well were calculated and shown in right panel. n = 4 wells for each group. (F) Expression levels of *SNORD15B* in CRC samples derived from TCGA. *SNORD15B* expression was normalized to endogenous *U6.* (G) Expression levels of *SNORD15B* in CRC tumors and peri-tumors. *SNORD15B* expression was normalized to endogenous *U6.* (H) Quantification of relative protein levels of Ilf2-binding splicing factors in Figure 4B. Values for *Snord15b*^−/−^ mice are presented as percentages of those for *Snord15b*^+/+^ mice. Data are shown as means ± SD. Statistical analysis was performed using unpaired two-tailed Student’s t-tests (F) and one-way ANOVA with Tukey's multiple comparisons testing (E); comparisons two groups for a single variable were performed via multiple unpaired Student's t-tests with Holm-Šidák correction (D, G and H). *P < 0.05, **P < 0.01, ***P < 0.001, ns, not significant.


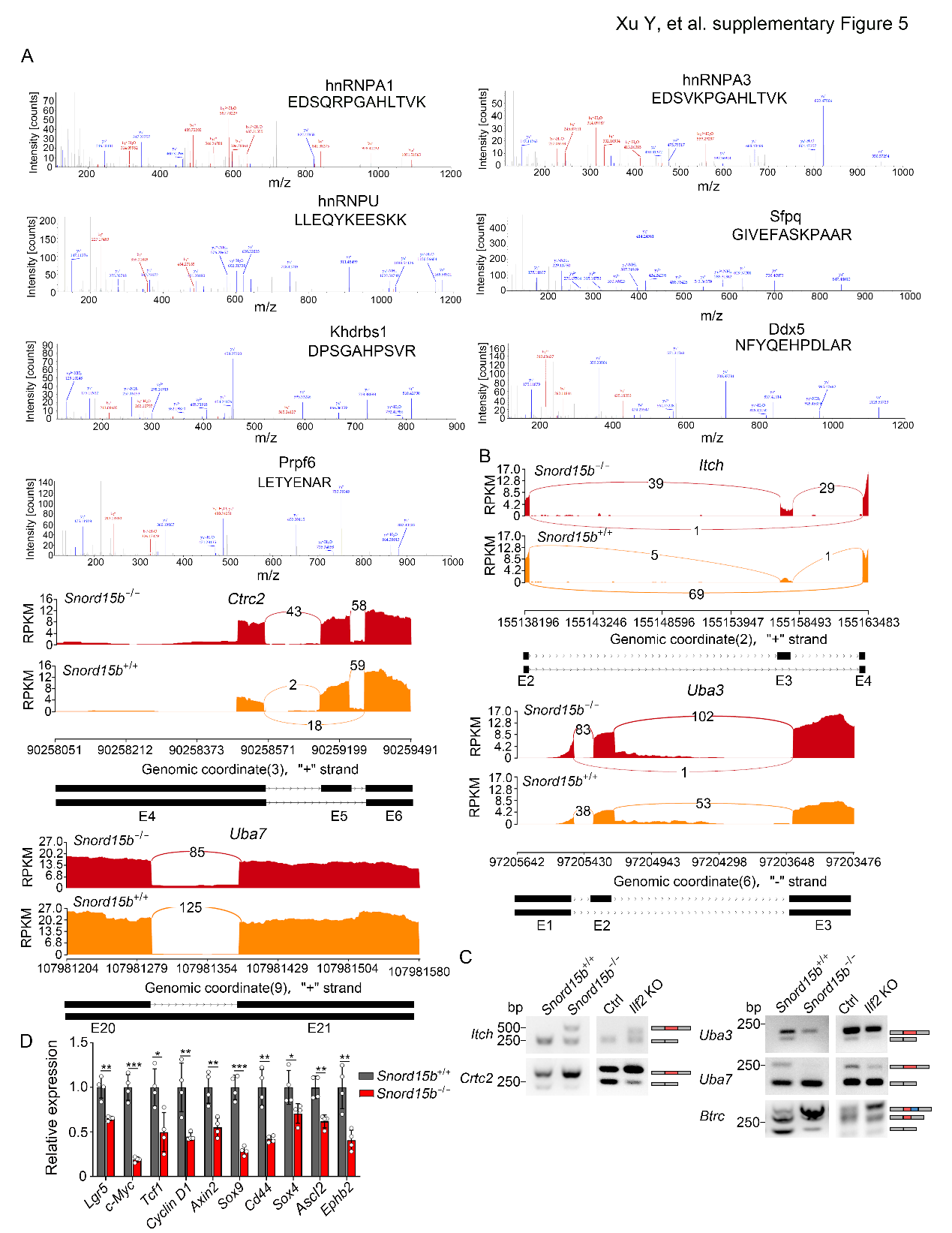


**Figure S5. Alternative splicing of genes associated with ubiquitin mediated proteolysis.** (A) MS profiles of hnRNPA1, hnRNPA3, hnRNPU, Sfpq, Khdbs1, Ddx5, and Prpf6, corresponding peptide sequences were listed on the top of diagram. (B) Representative Sashimi plots depicting alternative splicing pattern of *Itch*, *Crtc2*, *Uba3* and *Uba7* in *Snord15b*^+/+^ and *Snord15b*^−/−^ ISCs. Horizontal and vertical axis represents per-base expression and genomic coordinates, respectively. mRNA isoforms quantified was showed at bottom; black boxes and lines with arrow heads represent exons and introns, respectively. (C) Divergent AS events of genes associated with ubiquitin mediated proteolysis in Figure 4D were validated in *Snord15b* KO, *Ilf2* KO and their respective control intestinal organoids via semi-quantitative PCR. (D) Expression of Wnt target genes in *Snord15b*^+/+^ and *Snord15b*^−/−^ ISCs were examined by qRT-PCR. n = 4 independent experiments. Data are shown as the means ± SD. Data are shown as means ± SD. Comparisons two groups for a single variable were performed via multiple unpaired Student's t-tests with Holm-Šidák correction. *P < 0.05, **P < 0.01, ***P < 0.001.


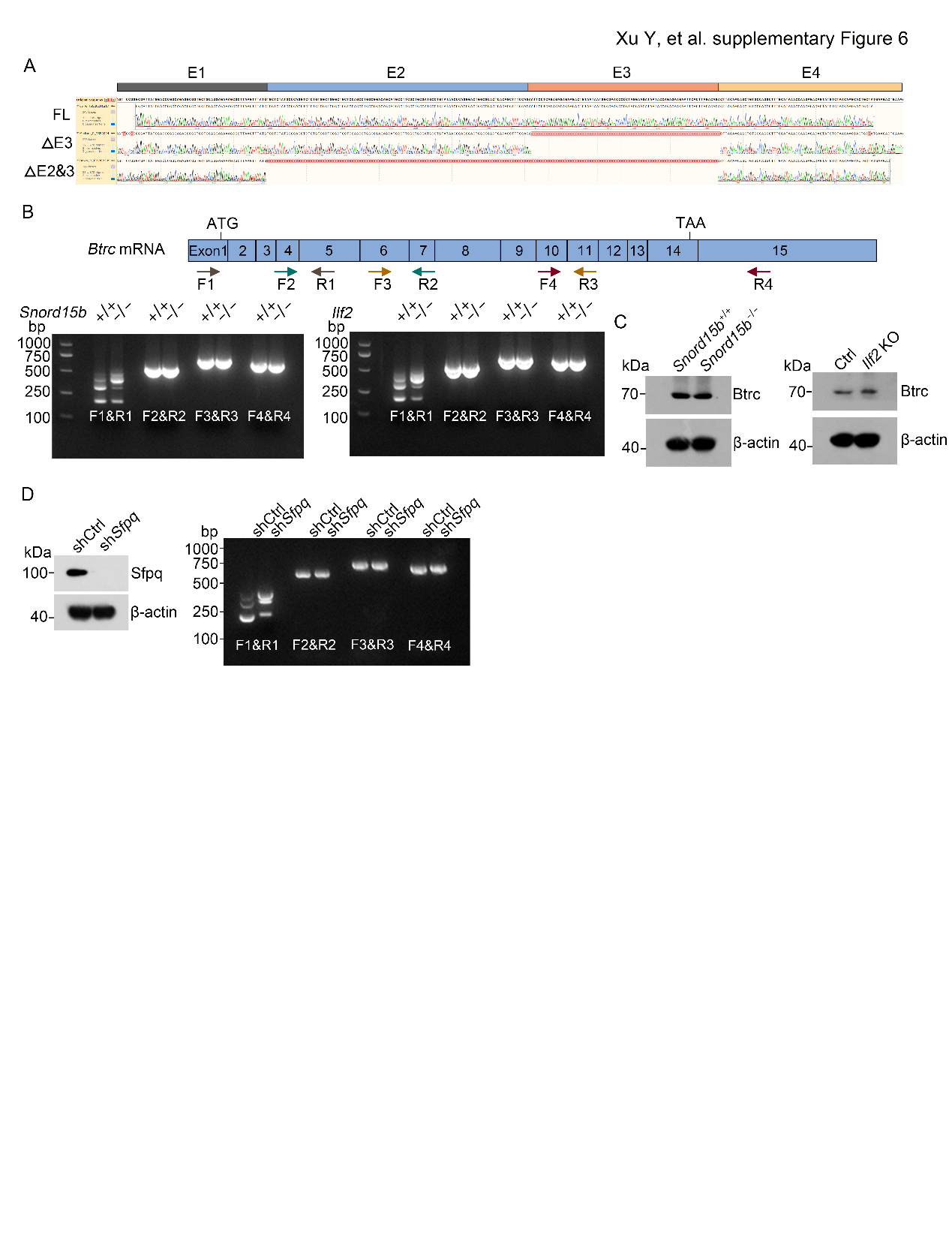


**Figure S6. Deletion of *Snord15b* or Ilf2 increases full length of Btrc.** (A) Validation of alternative splicing of *Btrc* exon 2 and exon 3 by PCR and sanger sequencing. (B) Analysis of alternative splicing events with four pairs of primers covering *Btrc* full-length CDS region. (C) Protein levels of Btrc in *Snord15b* KO, *Ilf2* KO and their corresponding control intestinal organoids were determined by Western blotting. (D) Knockdown efficiency of *Sfpq* in ISCs was examined by immunoblotting (left panel). n = 4 biologically independent samples. Analysis of alternative splicing events of *Btrc* with four pairs of primers as above (right panel).


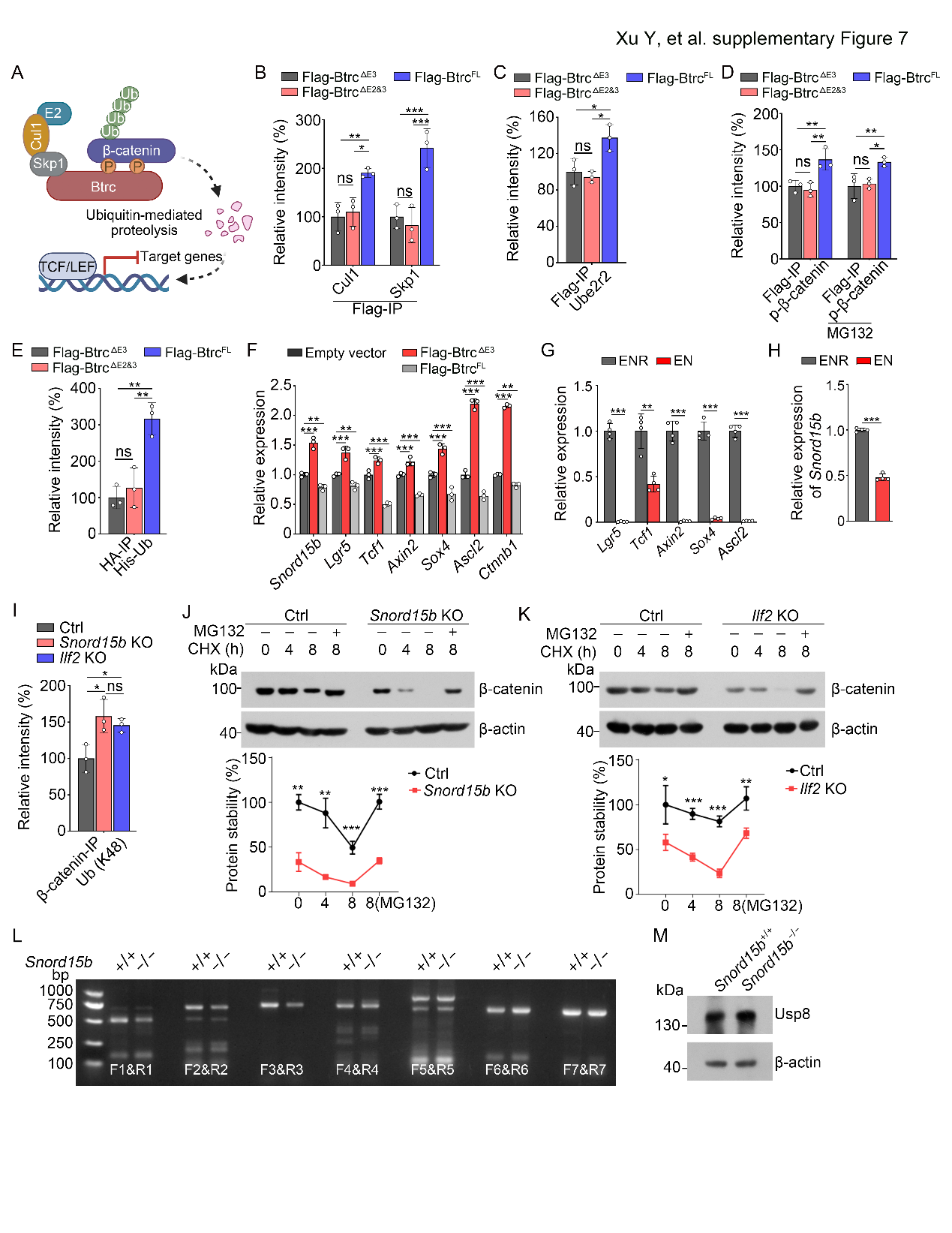


**Figure S7. Deletion of *Snord15b* or Ilf2 promotes the ubiquitination and degradation of β-catenin.** (A) A schematic of the Skp1-Cul1-Btrc ubiquitin protein ligase complex is shown. Skp1-Cul1-Btrc complex interacts with E2 ubiquitin-conjugating enzyme to mediate ubiquitination and subsequent proteasomal degradation of phosphorylated β-catenin, then decreased nuclear translocation of β-catenin. The schematic image was made using the BioRender software (https://www.biorender.com/). (B) Quantification of relative protein levels of Flag-immunoprecipitated Cul1 and Skp1 in Figure 5A. Values of cells expressing Flag-Btrc (△E2&3) and Flag-Btrc (FL) were presented as percentages compared to cells expressing Flag-Btrc (△E3). n = 3 independent experiments. (C) Quantification of relative protein levels of Flag-immunoprecipitated Ube2r2 in Figure 5C. Values of cells expressing Flag-Btrc (△E2&3) and Flag-Btrc (FL) were presented as percentages compared to cells cells expressing Flag-Btrc (△E3). n = 3 independent experiments. (D) Quantification of relative protein levels of Flag-immunoprecipitated β-catenin in Figure 5D. Values of cells expressing Flag-Btrc (△E2&3) and Flag-Btrc (FL) were presented compared to cells expressing Flag-Btrc (△E3). n = 3 independent experiments. (E) Quantification of relative protein levels of HA-immunoprecipitated His-Ubiquitin in Figure 5E. Values of cells expressing Flag-Btrc (△E2&3) and Flag-Btrc (FL) were presented as percentages compared to cells expressing Flag-Btrc (△E3). n = 3 independent experiments. (F) Expression of *Snord15b* and Wnt target genes in cells overexpressing short or full-length Btrc. n = 4 independent experiments. (G) Expression of Wnt target genes in organoids cultured in EN or ENR medium. n = 4 independent experiments. (H) Expression of *Snord15b* in organoids cultured in EN or ENR medium. n = 4 independent experiments. (I) Quantification of relative protein levels of ubiquitinated β-catenin immunoprecipitated by anti-β-catenin antibody in Figure 5F. Values of *Snord15b* KO and *Ilf2* KO cells were presented as percentages compared to Ctrl cells. n = 3 independent experiments. (J) *Snord15b*^+/+^ or *Snord15b*^−/−^ intestinal organoids were treated with CHX (20 μg/ml) and MG132 (10 μM). At different time points, equal amounts of cells were sampled and protein levels were analyzed by Western blotting (upper panel). Percentages of remaining protein amounts were calculated and normalized to *Snord15b*^+/+^ organoids (lower panel). n = 3 independent experiments. (K) *Ilf2* depleted or control intestinal organoids were treated with CHX (20 µg/ml) and MG132 (10 µM). At different time points, equal amounts of cells were sampled and protein levels were analyzed by Western blotting (left panel). Percentages of remaining protein amounts were calculated and normalized to control organoids (right panel). n = 3 independent experiments. (L) Analysis of alternative splicing events with seven pairs of primers covering *Usp8* full-length CDS region in *Snord15b*^+/+^ and *Snord15b*^−/−^ ISCs. (M) Protein levels of Usp8 in *Snord15b*^+/+^ and *Snord15b*^−/−^ ISCs were determined by Western blotting. Data are shown as the means ± SD. Statistical analysis was performed using unpaired two-tailed Student’s t-tests (H), one-way (C, E and I) and two-way ANOVA (B, D, and F) with Tukey's multiple comparisons testing; comparisons two groups for a single variable were performed via multiple unpaired Student's t-tests with Holm-Šidák correction (G, J and K). *P < 0.05, **P < 0.01, ***P < 0.001, ns, not significant.


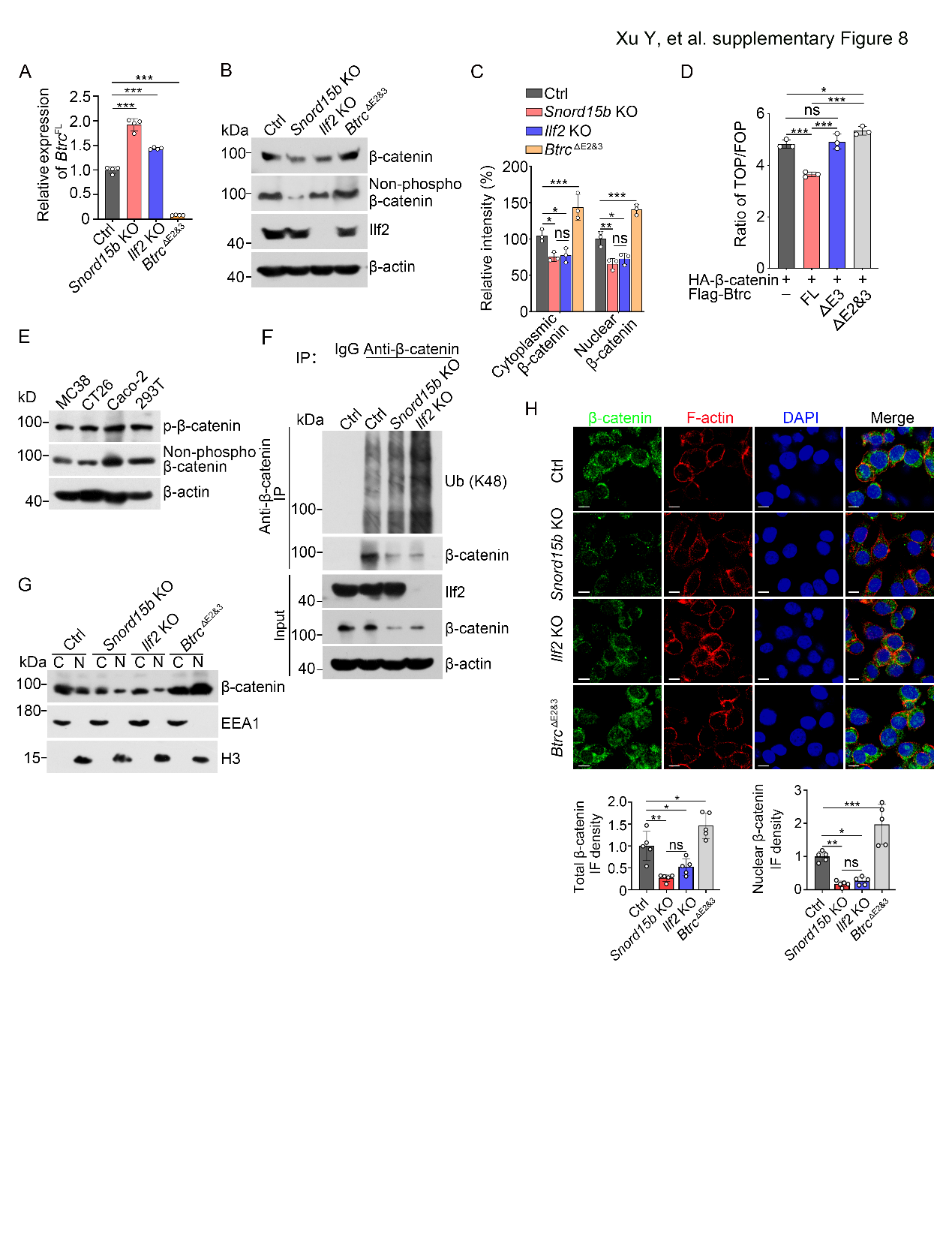


**Figure S8. Deletion of *Snord15b* or Ilf2 inhibits Wnt/β-catenin signaling activation.** (A) Knockout of exon 2 and 3 of *Btrc in* MC38 cells was validated via qRT-PCR. Data were normalized to endogenous *Actb*. n = 4 independent experiments. (B) Western blotting analysis of β-catenin and non-phospho β-catenin in WT, *Snord15b* KO ,*Ilf2* KO and *Btrc* (△E2&3) MC38 cells. Anti-Ilf2 antibody was used to validate the knockout of *Ilf2*. β-actin served as a control. (C) Quantification of relative protein levels of cytoplasmic and nuclear β-catenin in Figure 5G. Values of *Snord15b* KO, *Ilf2* KO and *Btrc* (△E2&3) cells were presented as percentages compared to control cells. n = 3 independent experiments. (D) Flag-tagged Btrc isoforms (△E3, △E2&3, FL) and HA-tagged β-catenin were cotransfected into HEK293T cells for 24 h, then indicated cells were transfected with TOP-flash or FOP-flash luciferase-reporter vectors. After 24 h, relative luciferase activity was calculated and TOP/FOP ratio was shown. n = 4 independent experiments. (E) Western blotting analysis of phosphor-β-catenin, β-catenin and non-phospho β-catenin in MC38, CT26, Caco-2 and 293T cells. (F) Lysates from WT, *Snord15b* KO and *Ilf2* KO CT26 cells were incubated with anti-β-catenin antibody for immunoprecipitation, followed by Western blotting with K48-linked specific ubiquitination antibody. (G) Western blotting analysis of β-catenin in nuclear and cytoplasmic fractionated extracts from WT, *Snord15b* KO, *Ilf2* KO and *Btrc* (△E2&3) CT26 cells. EEA1 and H3 are protein markers for cytoplasm and nucleus, respectively. (H) β-catenin distribution in nuclei and cytoplasm of WT, *Snord15b* KO, *Ilf2* KO and *Btrc* (△E2&3) CT26 cells was visualized via Immunofluorescence staining. Scale bars, 10 μm. Fluorescence intensity of total and nuclear β-catenin per field was calculated and compared with WT group. n = 5 fields for each group. Data are shown as the means ± SD. Statistical analysis was performed using one-way (A, D and G) and two-way ANOVA (C) with Tukey's multiple comparisons testing. *P < 0.05, **P < 0.01, ***P < 0.001, ns, not significant.


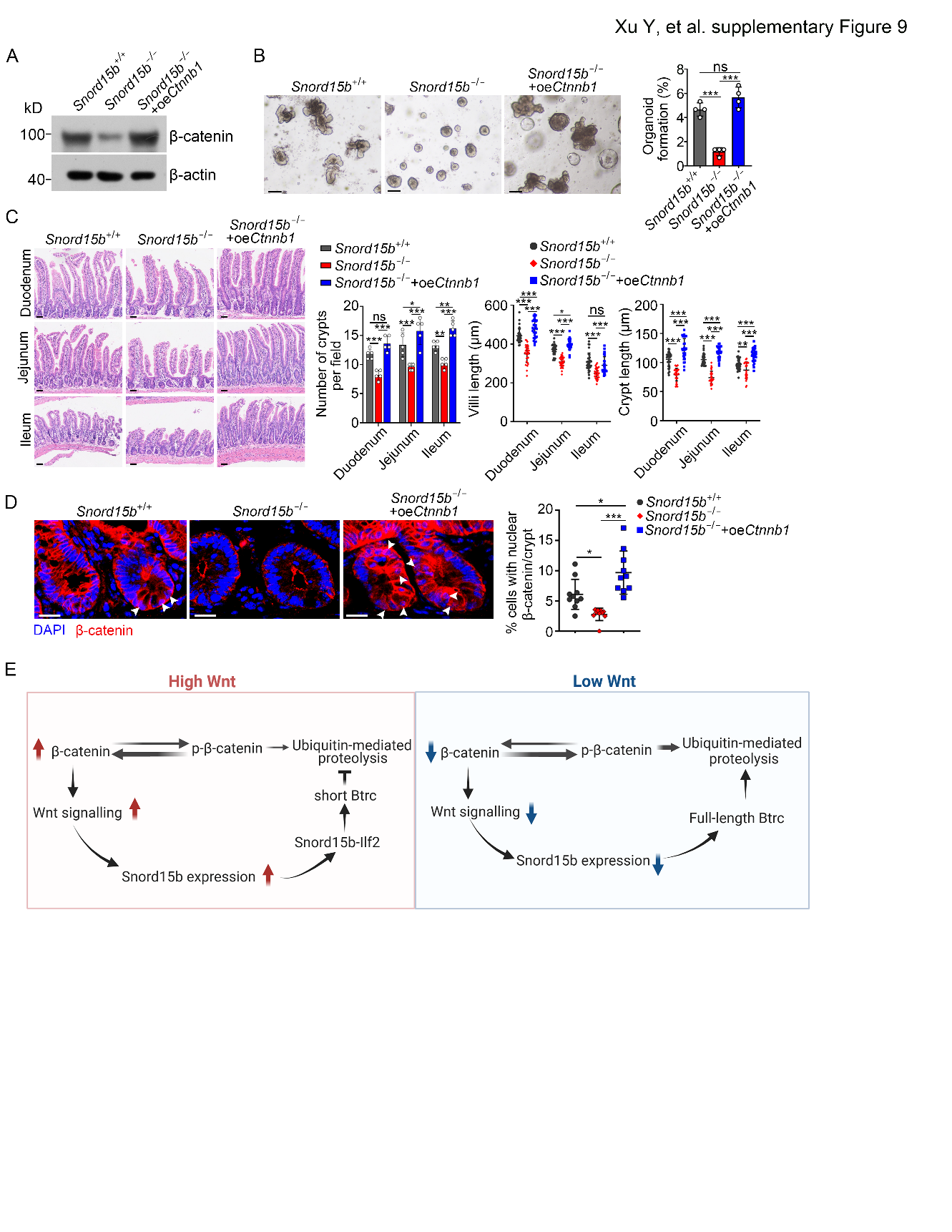


**Figure S9. β-catenin overexpression enhances stemness maintenance of ISCs.** (A-B) Overexpression of *Ctnnb1* was conducted in *Snord15b*^−/−^ mice via lentivirus infection. Crypts from indicated mice were isolated and organoids formation were performed. Expression levels of β-catenin in indicated organoids were detected by Western blotting. Scale bars, 100 μm. Ratios of organoid formation per well were calculated and shown. n = 4 wells for each group. (C) Representative H&E staining images of three small intestine regions: duodenum, jejunum and ileum from indicated mice. Scale bars, 50 μm. Lengths of villi and crypts, as well as crypt numbers per field are shown in right panel. n = 30 villi or crypts for length calculation. n = 5 fields for crypt number calculation. (D) Immunofluorescence visualization of β-catenin expression in intestinal crypts from indicated mice. Scale bars, 10 μm. In a crypt, proportions of cells with nuclear β-catenin expression among all crypt cells were calculated and shown in right panel. n = 10 crypts for each group. (E) In ISCs in the high-concentration Wnt niche, increased β-catenin activates the Wnt signaling pathway, which upregulates *Snord15b* expression and promotes *Snord15b*-Ilf2 mediated formation of short Btrc. This reduces phosphorylated catenin ubiquitination and degradation, keeping cellular phosphorylated catenin at a low level. Data are shown as means ± SD. Statistical analysis was performed using one-way (B and D) and two-way ANOVA with Tukey’s multiple comparisons test (C); *P < 0.05, **P < 0.01, ***P < 0.001, ns, not significant.

**Table S1. sgRNA sequences used in this study**

| Target gene | sgRNAs |
| --- | --- |
| *Snord15b* (up) | 5’- TCATCACTGAAGGGCACAGA -3’ |
| *Snord15b* (down) | 5’- TTAGAGGCATTTGTCTGAGA -3’ |
| *Ilf2* (up) | 5’- TGTATTTTTAGGGGTGACAG -3’ |
| *Ilf2* (down)  *Btrc_*E2&3 (up)  *Btrc_*E2&3 (down)  *Btrc* (down) | 5’- TGAAGTTGCCACTCTCACAC -3’  5’-CAGCCCAGCCACAGAGACCT-3’  5’-AGAGAAGAATTCACTTAGAC-3’  5’-AGAGAGTCCCTACCTGACGG-3’ |

sgRNAs were designed according to online available tool (<https://zlab.bio/guide-design-resources>), and purchased from Sangon Company.

**Table S2. shRNA sequences used in this study**

| Target gene | shRNAs |
| --- | --- |
| *Snord17* | 5’-GGTTCCATCTCACCAAGAT-3’ |
| *Snord55* | 5’-GATGATGACACCTGGGTAT-3’ |
| *Snora20* | 5’-GCGGTTATACGCATGGGAT-3’ |
| *Snord15b*  *Snord8*  *Snord83b*  *Snord66*  *Snora61*  *Snord88a*  *Snord14a*  *SNORD15B*  *ILF2*  *Sfpq* | 5’-GACACGATGACGAGTCAGAAT-3’  5’-GCAGTATTGTCCTGGTTTG-3’  5’-GAGAGAGACTGAGGAATTA-3’  5’-GCTGATGACTTCTGTCAGT-3’  5’-GGATCTTGACGCTGGTCTT-3’  5’-GCTCTGACCATCCTGAAGA-3’  5’-GGACTTTCCATGTTGGGTT-3’  5’-GATTTAGAGGCATTTGTCTGA-3’  5’-GGACATTTGAAGTGCAAAT-3’  5’-GAACCACTTGAACAGTTA-3’ |

shRNAs were designed according to online available tool (https://biosettia.com/support/shrna-designer/), and purchased from Sangon Company.

**Table S3. Primer sequences used for qPCR**

| Gene | Sequences |
| --- | --- |
| *Snord17* (Forward)  *Snord17* (Reverse)  *Snord55* (Forward)  *Snord55* (Reverse)  *Snora20* (Forward)  *Snora20* (Reverse)  *Snord15b* #1 (Forward)  *Snord15b* #1 (Reverse)  *Snord15b* #2 (Forward)  *Snord15b* #2 (Reverse)  *Snord15b* #3 (Forward)  *Snord15b* #3 (Reverse)  *Snord15a* (Forward)  *Snord15a* (Reverse)  *Snord8* (Forward)  *Snord8* (Reverse)  *Snord83b* (Forward)  *Snord83b* (Reverse)  *Snord66* (Forward)  *Snord66* (Reverse)  *Snora61* (Forward)  *Snora61* (Reverse)  *Snord88a* (Forward)  *Snord88a* (Reverse)  *Snord14a* (Forward)  *Snord14a* (Reverse)  *Lgr5* (Forward)  *Lgr5* (Reverse)  *c-Myc* (Forward)  *c-Myc* (Reverse)  *Tcf1* (Forward)  *Tcf1* (Reverse)  *Cyclin D1* (Forward)  *Cyclin D1* (Reverse)  *Axin2* (Forward)  *Axin2* (Reverse)  *Sox9* (Forward)  *Sox9* (Reverse)  *Cd44* (Forward)  *Cd44* (Reverse)  *Sox4* (Forward)  *Sox4* (Reverse)  *Ascl2* (Forward)  *Ascl2* (Reverse)  *Ephb2* (Forward)  *Ephb2* (Reverse)  *Btrc* (Forward)  *Btrc* (Reverse)  *Snord15a* (Forward)  *Snord15a* (Reverse)  *Rps3* #1 (Forward)  *Rps3* #1 (Reverse)  *Rps3* #2 (Forward)  *Rps3* #2 (Reverse)  *Rps3* #3 (Forward)  *Rps3* #3 (Reverse)  *U6* (Forward)  *U6* (Reverse)  *Gapdh* (Forward)  *Gapdh* (Reverse)  *Actb* (Forward)  *Actb* (Reverse)  *Oct4* (Forward)  *Oct4* (Reverse)  *Ilf2* (Forward)  *Ilf2* (Reverse)  *SNORD15B* (Forward)  *SNORD15B* (Reverse)  *ILF2* (Forward)  *ILF2* (Reverse)  2’-O-Me (F_U_)  2’-O-Me (F_D_)  2’-O-Me (R) | 5’-CTGACCTTCTTCCCAGTCTCG-3’  5’-CCTGCTGACACTAGCCATTC-3’  5’-GGATGATGACACCTGGGTATGC-3’  5’-ACGCTCAGCTCTCCAAGGTT-3’  5’-TGTCAGTGATAGGAGCGGTT-3’  5’-ACCATGTACGAGAGACAAAACA-3’  5’-TTCAGTGATGACACGATGACGA-3’  5’-CTCAGACAAATGCCTCTAAATCAA-3’  5’-TCAGAATGGCCACGTCTTGC-3’  5’-CACTTTTGCCAAGGGAACCA-3’  5’-TCAGTGATGACACGATGACGAG-3’  5’-CCACAGAACATGGCACTGAC-3’  5’-TCACTATGCTGCGTTCTGTGG-3’  5’-AGACAAATGCCTCTTGGTCATC-3’  5’-TCCCGATGATGAGCTGCC-3’  5’-AACTCACTGGCACCCAAACC-3’  5’-GCTGTTCTGTGATGAGGCTCT-3’  5’-GAAGGAAGGCAACAGGGAATAA-3’  5’-TTCCGCTGATGACTTCTGTC-3’  5’-TCCTCAGGTCCTCAATCCCA-3’  5’-CCTCCGGTTTCCCTTTCCC-3’  5’-CTTCAGGGTGCTCTTCCTCC-3’  5’-GGACCCCCTTGATGTCCA-3’  5’-CCAGGGTGTCAAAGGTCCC-3’  5’-TGGTGTTCCAACATTCGCAGTT-3’  5’-CAGACATCCAAGGAAGGTCAA-3’  5’-TTGAGGAAGACCTGAAGGC-3’  5’-TCCACTACCGCGATTACC-3’  5’-TAGTGCTGCATGAGGAGACA-3’  5’-CATCAATTTCTTCCTCATCTTC-3’  5’-CTGCCTGCTCACAGTTCC-3’  5’-GGCTCCAGGCCTGTGG-3’  5’-ATTGTGCCATCCATGCG-3’  5’-TAGATGCACAACTTCTCGGC-3’  5’-TGCCGACCTCAAGTGCA-3’  5’-ACGCTACTGTCCGTCATGG-3’  5’-ACAACGCGGAGCTCAGC-3’  5’-GAGTCGGCTTGCAGCG-3’  5’-CATCGAGAAGAGCACCCCAG-3’  5’-TGAGTGCACAGTTGAGGCAA-3’  5’-GGGGAACTCTGCCGGTAAC-3’  5’-TGCAACAGACCGGCATGAATA-3’  5’-AAGCACACCTTGACTGGTACG-3’  5’-AAGTGGACGTTTGCACCTTCA-3’  5’-CGTGAAAGTGGACACCATTG-3’  5’-CCAAGTAGAAGCCAGCCTTG-3’  5’-CTCTGTGGCTGGGCTGCTCCAG-3’  5’-GGGGTTCGCCATTATTACAGTC-3’  5’-TCACTATGCTGCGTTCTGTGG-3’  5’-AGACAAATGCCTCTTGGTCATC-3’  5’-ATGGCGGTGCAGATTTCCAA-3’  5’-GTAACTCGGACTTCAACTCCAG-3’  5’-CCCCATCTCCGAACAGAAGG-3’  5’-TGGACACAATCCAGTCAGGC-3’  5’-GCTGGCTGAAGATGGCTACT-3’  5’-TCTGATCCGACGACCCTTCT-3’  5’-GCTTCGGCAGCACATATACTAAAAT-3’  5’-CGCTTCACGAATTTGCGTGTCAT-3’  5’-AGGTCGGTGTGAACGGATTTG-3’  5’-TGTAGACCATGTAGTTGAGGTCA-3’  5’-GGCTGTATTCCCCTCCATCG-3’  5’-CCAGTTGGTAACAATGCCATGT-3’  5’-AGAGGATCACCTTGGGGTACA-3’  5’-CGAAGCGACAGATGGTGGTC-3’  5’-CCTGGGGAACAAAGTCGTGG-3’  5’-TGAGAATTTTCACCGTAGCATCA-3’  5’-CAGTGATGACACGATGACGAG-3’  5’-GCACCACAGAACATGGCACT-3’  5’-TGGGGAACAAAGTCGTGGAA-3’  5’-GCATTTTCCTCGAACCAGCG-3’  5’-CGGGTGTTGACGCGATGTGATT-3’  5’-GTCATCTAATTAGTGACGCGCAT-3’  5’-CGTTCCCTTGCGTGGTTTCG-3’ |

**Table S4. Primer sequences used for semi-quantitative PCR**

| Gene | Sequences |
| --- | --- |
| *Btrc* #1 (Forward) | 5’-CAGTAGTCCGGGCGATTATGGA-3’ |
| *Btrc* #1 (Reverse)  *Btrc* #2 (Forward) | 5’-TTGGGCACAATCATGCTGGAAG-3’  5’-AGGCTTTGCATAAACCAAGA-3’ |
| *Btrc #2* (Reverse)  *Btrc* #3 (Forward)  *Btrc #3* (Reverse)  *Btrc* #4 (Forward)  *Btrc #4* (Reverse) | 5’-TAAAAGGAGTTGGGAGGAGC-3’  5’-CCTGGCAGAGCGCAGAGGCT-3’  5’-CCACGCTTGTGCCCATTTAG-3’  5’-AGCTGCGGTCAATGTTGTAG-3’  5’-GATTGTTGTTCATCCTGGTCTT-3’ |
| *Itch* (Forward)  *Itch* (Reverse)  *Crtc* (Forward)  *Crtc* (Reverse)  *Uba3* (Forward)  *Uba3* (Reverse)  *Uba7* (Forward)  *Uba7* (Reverse) | 5’-GAAGTAGCTCTAGTCGCTGTAGT-3’  5’-TGAAGTTGAGATTTCATGGTCA -3’  5’-GGCTGTGGCCTGGCTGAGTTCC-3’  5’-CGTTCTCCTCAATAGCAGGGACT-3’  5’-AAGAGGCGGGGAACAATATG-3’  5’-GAAACATCTATAGTGTCCATGTCTAT-3’  5’-GATGATGACAGCAACTTCCATG-3’  5’-GCCCAGTAAGCCTGCCACAACT-3’ |
